# Structural Characterization of the Essential Cell Division Protein FtsE and Its Interaction with FtsX in Streptococcus pneumoniae

**DOI:** 10.1128/mBio.01488-20

**Published:** 2020-09-01

**Authors:** Martin Alcorlo, Daniel Straume, Joe Lutkenhaus, Leiv Sigve Håvarstein, Juan A. Hermoso

**Affiliations:** a Department of Crystallography and Structural Biology, Institute of Physical-Chemistry “Rocasolano”, Spanish National Research Council (CSIC), Madrid, Spain; b Department of Chemistry, Biotechnology, and Food Science, Norwegian University of Life Sciences, Ås, Norway; c Department of Microbiology, Molecular Genetics, and Immunology, University of Kansas Medical Center, Kansas City, Kansas, USA; Harvard Medical School; Fred Hutchinson Cancer Research Center

**Keywords:** ABC transporters, ATPase, FtsEX, *Streptococcus pneumoniae*, X-ray crystallography, cell division, peptidoglycan hydrolases, protein-protein interactions

## Abstract

FtsEX is a membrane complex widely conserved across diverse bacterial genera and involved in critical processes such as recruitment of division proteins and in spatial and temporal regulation of muralytic activity during cell division or sporulation. FtsEX is a member of the ABC transporter superfamily. The component FtsX is an integral membrane protein, whereas FtsE is an ATPase and is required for the transmission of a conformational signal from the cytosol through the membrane to regulate the activity of cell wall hydrolases in the periplasm. Both proteins are essential in the major human respiratory pathogenic bacterium Streptococcus pneumoniae, and FtsX interacts with the modular peptidoglycan hydrolase PcsB at the septum. Here, we report high-resolution structures of pneumococcal FtsE bound to different nucleotides. Structural analysis revealed that FtsE contains all the conserved structural motifs associated with ATPase activity and afforded interpretation of the *in vivo* dimeric arrangement in both the ADP and ATP states. Interestingly, three specific FtsE regions with high structural plasticity were identified that shape the cavity in which the cytosolic region of FtsX would be inserted. The residues corresponding to the FtsX coupling helix, responsible for contacting FtsE, were identified and validated by *in vivo* mutagenesis studies showing that this interaction is essential for cell growth and proper morphology.

## Abstract (precis)

Bacterial cell division is a central process that requires exquisite orchestration of both the cell wall biosynthetic and lytic machineries. The essential membrane complex FtsEX, widely conserved across bacteria, plays a central role by recruiting proteins to the divisome apparatus and by regulating periplasmic muralytic activity from the cytosol. FtsEX is a member of the type VII family of the ABC-superfamily, but instead of being a transporter, it couples the ATP hydrolysis catalyzed by FtsE to mechanically transduce a conformational signal that provokes the activation of peptidoglycan (PG) hydrolases. So far, no structural information is available for FtsE. Here, we provide the structural characterization of FtsE, confirming its ATPase nature and revealing regions with high structural plasticity which are key for FtsE binding to FtsX. The complementary binding region in FtsX has also been identified and validated *in vivo*. Our results provide evidence on how the difference between the ATP/ADP-bound states in FtsE would dramatically alter the interaction of FtsEX with the PG hydrolase PcsB in pneumococcal division.

## INTRODUCTION

In order to divide, bacteria must synthesize a new septal cell wall in which the main component is peptidoglycan (PG). This newly synthetized PG must be split down the middle by one or more murein hydrolases to separate the resulting daughter cells. These biosynthetic and hydrolytic processes must be tightly coordinated and regulated to ensure bacterial survival. Although the regulation of these processes by PG hydrolases is not well understood, a principle that has emerged from recent work is that division PG hydrolases are usually autoinhibited (1–4) and require interactions with regulatory proteins, such as EnvC and NlpD in Escherichia coli, to activate synchronized cleavage of the PG cell wall. Lytic activity is thus regulated, spatially and temporally, by coupling their activation to the assembly of the cytokinetic ring (5) through the ATP-binding cassette (ABC) transporter family complex FtsEX. This membrane complex is widely conserved across diverse bacterial genera and functions as a dimer with FtsE monomers in the cytoplasm and FtsX monomers in the inner membrane (6). In E. coli, FtsE is one of the earliest proteins that assembles along with FtsX at the midcell site during cell division. FtsEX directly participates in this critical process by promoting septal-ring assembly in low-osmolarity medium (7). Indeed, FtsEX acts on FtsA to promote divisome assembly, and continual ATP hydrolysis by FtsE is needed for the divisome to synthesize septal PG (8). It is worth mentioning that binding of FtsE to FtsX is independent of the nucleotide (ATP or ADP), as FtsE ATP-binding or hydrolysis mutants still localize to septal rings *in vivo* when coexpressed with FtsX (9). In addition, FtsEX directly recruits periplasmic EnvC to the septum via a periplasmic loop in FtsX. Interestingly, ATPase-defective FtsEX variants still recruit EnvC to the septum but fail to promote cell separation in E. coli (10). In the divisome of Gram-negative bacteria, FtsE also interacts with FtsZ (11), linking the Z ring to the transmembrane protein FtsX, and it has been suggested that FtsEX may function as a membrane anchor for FtsZ utilizing ATP binding and hydrolysis to regulate Z-ring constriction (7, 11). Very recently, it was shown in E. coli that recruitment of FtsE and of FtsX are codependent, suggesting that the FtsEX complex forms through FtsE interacting with the conserved tail of FtsZ (12).

**FIG 1:**
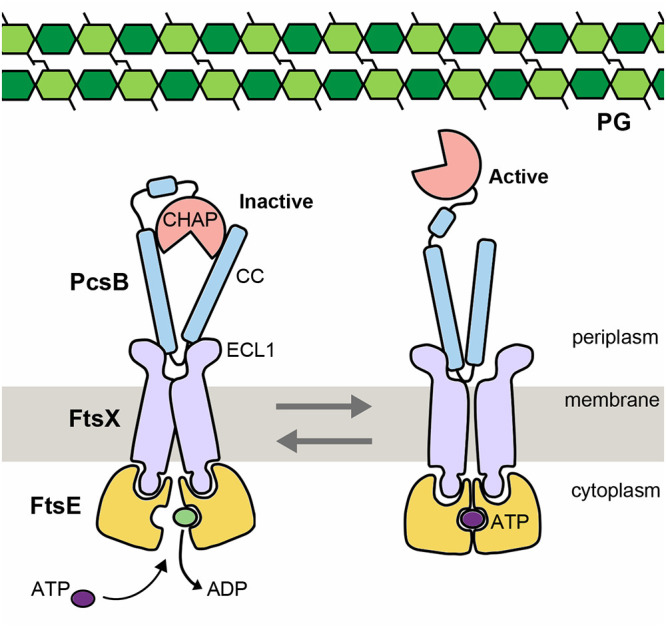
Scheme showing regulation of PG hydrolase PcsB by the FtsEX system in S. pneumoniae. PcsB binds to the large extracellular loop (ECL1) of FtsX independently of ATP. This binding occurs through its coiled coil (CC) domain. The stoichiometry between FtsX and PcsB is unknown; here, it is represented as 2:1. The catalytic domain of PcsB (CHAP) is generally autoinhibited but can be activated by ATP hydrolysis of FtsEX. ATP binding leads to conformational changes in FtsEX which impact CC, leading to activation of CHAP.

The general organization of FtsEX complexes resembles an ABC transporter (7, 9, 13). This family of proteins is widespread in all forms of life and is characterized by having two copies of both the nucleotide-binding domain (NBD) and the transmembrane domain (TMD). The NBD forms dimers, which can adopt open or closed conformations. NBD are coupled to the TMD through the so-called coupling helix at the NBD-TMD interface (reviewed in reference 14). ATP hydrolysis in the NBD drives conformational changes that are transmitted through the coupling helices in the TMD, resulting in alternating access from inside and outside the cell for unidirectional transport of a variety of compounds across the lipid bilayer (15). These proteins play a causative role in a number of human diseases, notably, cystic fibrosis (16), and multidrug resistance by tumor cells and microbial pathogens (17). However, although FtsEX is considered a member of the type VII family of the ABC superfamily (18), there is no evidence that FtsEX acts as a transporter. Instead, members of this family are not transporters at all but couple ATP hydrolysis in the cytoplasm with transmembrane conformational changes to perform mechanical work in the periplasm. Thus, the FtsEX is believed to transduce a conformational signal that would couple cell division to activate bound PG hydrolases, which vary in different bacterial species (1, 10, 19, 20). In Streptococcus pneumoniae, FtsX interacts with the PG hydrolase PcsB (20–24), and ATPase activity by FtsE (reported in reference 25) would trigger activation of PcsB (Fig. 1). PcsB is a modular protein containing a catalytic CHAP (cysteine, histidine-dependent amidohydrolases/peptidases) domain and a V-shaped coiled-coil (CC) domain (Fig. 1). The crystal structure of full-length PcsB revealed that it adopts a dimeric state in which the V-shaped CC domain of each monomer acts as a pair of molecular tweezers that clamps the catalytic CHAP domain of each partner in an inactive configuration (21). Solution studies pointed out that inactive conformation is also present at low concentrations, in which monomers of PcsB would present the CHAP domain inserted in its own CC domain (21).

FtsE, FtsX, and PcsB are essential for growth in some serotype strains of S. pneumoniae (24, 26, 27), and their absence causes severely diminished growth and cell morphology defects in others (28, 29). In strains where FtsEX-PcsB is essential, residue substitutions that inactivate FtsE are not tolerated (24). In E. coli, Neisseria gonorrhoeae, Aeromonas hydrophila, and Flavobacterium johnsoniae, *ftsE* and/or *ftsX* mutants exhibit division defects, indicating that FtsEX’s function in cell division is conserved in these organisms (30–33). In contrast, the FtsEX of B. subtilis has no obvious role in cell division but instead controls the peptidoglycan hydrolase CwlO, which plays an important role in cell wall expansion during cell elongation (19, 34, 35) and regulates entry into sporulation (36). FtsEX was recently reported to be involved in other biological processes such as control of both the early and late stages of cytokinesis in the alphaproteobacterium Caulobacter crescentus (37) or control of the activity of autolysin CwlO to regulate biofilm formation in Bacillus velezensis strain SQR9, a plant-beneficial rhizobacterium (38).

S. pneumoniae and most other Gram-positive bacteria synthesize a complete septal cross wall before cell separation takes place at the end of the cell cycle. In Gram-negative bacteria, on the other hand, synthesis and splitting of the septal cross wall and invagination of the outer membrane occur in tandem (39). Hence, the constrictive mode of cell division displayed by Gram-negative bacteria requires a tight coupling between peptidoglycan synthesis and hydrolysis during septation. In E. coli, FtsEX, which localizes to the Z ring, has been shown to play an important role in this process (12). In this Gram-negative species, FtsEX is involved in assembly of the divisome, activation and regulation of peptidoglycan synthesis, and recruitment and activation of cross wall splitting hydrolases (6). In S. pneumoniae, synthesis and splitting of the septal cross wall are separate in time and space. The separation of daughter cells takes place at the end of the cell cycle, well after the synthesis of the septal cross wall has been completed. Hence, it is highly unlikely that the FtsEX-PcsB complex is associated with the Z ring and the core divisome in S. pneumoniae. We previously reported that pneumococci form very long chains when PBP2b, a key component of the elongasome, is depleted. Furthermore, transmission electron microscopy analysis of these cells revealed that their septal cross walls have not been cleaved (40). Thus, when the elongasome is no longer functional, the separation of daughter cells mediated by the FtsEX-PcsB complex does not take place. This observation shows that the elongasome must be properly assembled before the FtsEX-PcsB complex becomes active, suggesting that the signal that triggers cross wall splitting and daughter cell separation in S. pneumoniae is linked to elongasome assembly and the initiation of lateral peptidoglycan synthesis.

Despite the relevant biological roles exerted by FtsEX, no structural precedent for any FtsE is available to date. Here, we provide the first three-dimensional (3D) structure of FtsE from the notorious pathogen S. pneumoniae, and we show that it presents all the conserved structural motifs of the NBD members of the ABC transporter family. We obtained high-resolution FtsE structures from two different crystal forms revealing an unexpectedly high conformational plasticity. The results are discussed in the context of the complex that FtsE forms with the transmembrane protein FtsX. In addition, we identified the FtsX region recognized by FtsE in the pneumococcal complex. This finding was further validated *in vivo*, allowing us to propose a structural model accounting for the interaction between the two proteins.

## RESULTS

### Three-dimensional structure of FtsE from Streptococcus pneumoniae.

**FIG 2:**
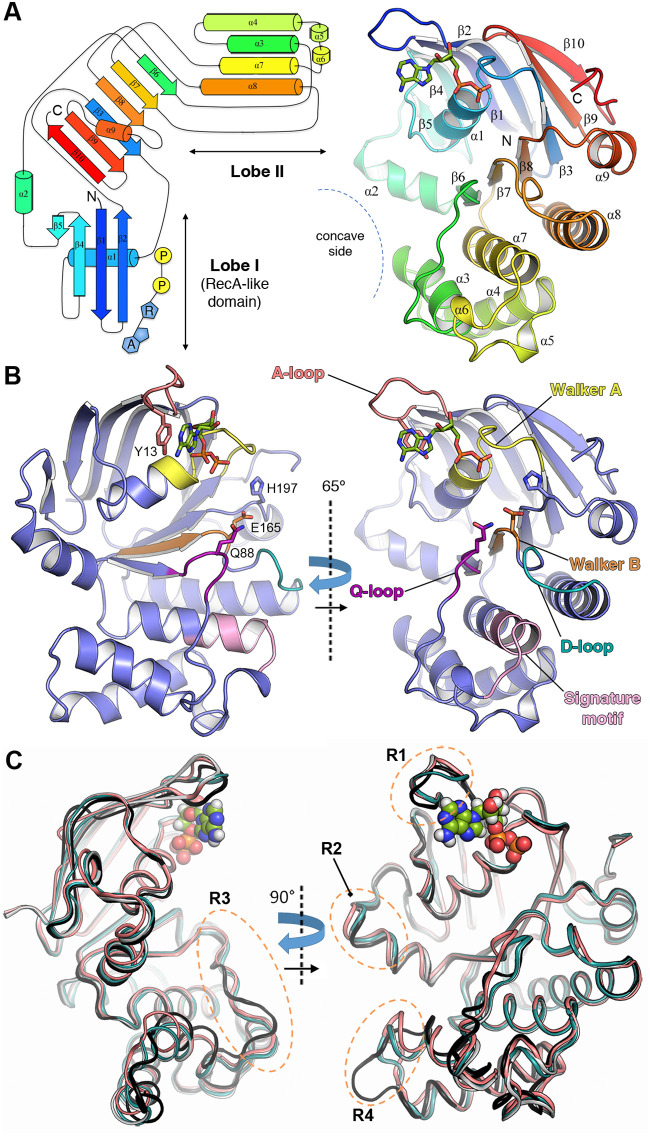
Three-dimensional structure of FtsE in complex with ADP and structural plasticity in the FtsE monomer. (A) (Left) Topological diagram of the secondary structure of FtsE following the rainbow color code. Cylinders and arrows represent α-helices and β-strands, respectively. ADP is shown in blue and yellow (A, adenine; R, ribose; P, phosphate group). (Right) Overall ribbon representation of the FtsE monomer structure. Secondary structure elements are indicated and numbered following the same color code as indicated in panel A. N, N terminus; C, C terminus. The ADP molecule is depicted as sticks. (B) Locations of the conserved and functionally critical motifs present in the FtsE structure, including Walker A/B, A-loop, D-loop, Q-loop, and signature motif (colored in yellow/orange, red, cyan, magenta, and pink, respectively). ADP, the catalytic glutamate (E165), the conserved glutamine (Q88) from the Q-loop, and switch histidine (H197) are shown as sticks. The FtsE cartoon structure is displayed in two orientations at 65° of each other. (C) Structural superposition of the four independent FtsE monomers in complex with ADP (depicted as spheres). The protein structures are displayed in cartoon ovals at two orientations at 90° of each other. Monomer P 1 is colored in salmon red, M1 is colored in black, M2 is colored in cyan, and M3 is colored in pale blue. The four regions with main structural variability are indicated with dashed orange ellipsoids and labeled R1, R2, R3, and R4 (see text for details).

FtsE was purified to homogeneity and concentrated up to 8 mg/ml (Fig. S1A) in a solution containing 25 mM Tris-HCl (pH 7.5), 2 mM dithiothreitol (DTT), and 100 mM NaCl. Two different crystal forms in complex with ADP were obtained in the same crystallization conditions belonging to two different space groups, P 1 and P 2_1_ (see Materials and Methods). P 1 crystals contain one molecule per asymmetric unit and diffract up to 1.36 Å resolution (Fig. S1C), while P 2_1_ crystals contain three molecules per asymmetric unit and diffract up to 1.57 Å resolution (Table 1). The FtsE structure was solved by *de novo* phasing with ARCIMBOLDO (41) (see Materials and Methods). In both P 1 and P 2_1_, the resulting structures correspond to monomeric FtsE and provided excellent electron density for all 230 amino acid residues of the protein (Fig. S1D). The FtsE structure has overall dimensions of 49.6 by 41.5 by 27.0 Å comprising two thick lobes (lobe I and II) grouped together resembling an L shape with convex and concave sides (Fig. 2A), as previously reported for other ATPases (42). Unless otherwise indicated, the description of the monomer structure provided here is for the highest-resolution crystal form belonging to space group P 1. In FtsE, the ATP-binding pocket is located close to the end of lobe I, a subdomain which is present in many other ATPases (43). FtsE lobe I (residues 1 to 87, 159 to 164, and 191 to 230) consists of a combination of α and β structures (α1 and α2 and β-sheet I, including strands β1-β2 and β4-β5) mainly formed by the packing of α1 against the antiparallel β-sheet I. Lobe II (residues 88 to 158 and 165 to 190) is composed of only α helices (α3 to α8). Lobes I and II are connected by the central β-sheet II that comprises strands β3, β6-β7-β8-β9, and β10 (Fig. 2A).

**TABLE 1:** Crystallographic data collection and refinement statistics^*a*^

Parameter	Value		
	FtsE-ADP	FtsE-ADP	FtsE–AMP-PNP
Data collection			
Wavelength (Å)	0.97923	0.96862	0.97934
Space group	P 1	P 2_1_	P 2_1_
Cell dimensions			
*a, b, c* (Å)	33.39, 36.71, 41.11	33.05, 118.54, 82.37	33.07, 117.90, 81.48
α, β, γ (º)	105.27, 95.69, 99.69	90, 97.55, 90	90, 98.11, 90
Temp (K)	100	100	100
X-ray source	Synchrotron	Synchrotron	Synchrotron
Resolution range (Å)	39.20–(1.38–1.36)	47.97–(1.60–1.57)	47.60–(2.49–2.40)
No. of unique reflections	37,746 (1,797)	86,937 (4,252)	23,969 (2,528)
Completeness (%)	95.80 (92.50)	99.50 (99.50)	99.00 (99.80)
Multiplicity	8.6 (8.5)	5.0 (5.0)	5.8 (5.7)
*R_merge_*^*b*^	0.074 (0.932)	0.081 (0.817)	0.100 (0.550)
*R_pim_*^*c*^	0.026 (0.337)	0.039 (0.396)	0.045 (0.252)
*<I/σ(I)>*	16.5 (2.3)	11.7 (2.1)	10.6 (2.8)
CC1/2	0.99 (0.84)	0.99 (0.56)	0.99 (0.91)
Refinement			
Resolution range (Å)	34.68–1.36	35.57–1.57	47.60–2.4
*R_work_/R_free_*^*d*^	0.1780/0.1932	0.1826/0.2129	0.1892/0.2490
No. of atoms			
Protein	1,835	5,442	5,535
Water	298	645	52
Ligand	27	135	89
RMSD			
Bond length (Å)	0.006	0.009	0.007
Bond angles (°)	0.99	1.17	1.13
Ramachandran analysis			
Favored/outliers (%)	98.68/0	98.38/0.15	97.33/0
Monomers per AU	1	3	3
Average B-factor	17.65	22.81	51.02
Macromolecules	16.41	21.64	50.92
Ligands	12.03	33.65	61.35
Solvent	25.79	30.37	43.62
PDB code	6Z4W	6Z63	6Z67

a Values between parentheses correspond to the highest resolution shells.

b *R*_merge_ = Σ_hkl_ Σ_i_ | I_i_(hkl) – [I(hkl)] |/Σ_hkl_Σ_i_I_i_(hkl), where Σ_i_*I*_i_(hkl) is the *i*-th measurement of reflection hkl and [I(hkl)] is the weighted mean of all measurements.

c *R*_pim_ = Σ_hkl_[1/(*N* – 1)]^1/2^ Σ_i_ | *I*_i_(hkl) – [*I*(hkl)] |/Σ_hkl_Σ_i_*I*_i_(hkl), where Σ_i_*I*_i_(hkl) is the *i*-th measurement of reflection hkl, [*I*(hkl)] is the weighted mean of all measurements, and *N* is the redundancy for the hkl reflection.

d *R*_work_/*R*_free_ = Σ_hkl_| *F*_o_ – *F*_c_ |/Σ_hkl_ | *F*_o_ |, where *F*_c_ is the calculated and *F*_o_ is the observed structure factor amplitude of reflection hkl for the working/free (5%) set, respectively.

10.1128/mBio.01488-20.1 FIG S1 (A) SDS-PAGE (12%) analysis of the eluted pure FtsE protein after His-tag removal with TEV protease. Molecular weight markers (kDa) are indicated for the left lane. (B) FtsE crystals belonging to space group P 2_1_ diffracted up to 1.57 Å resolution. Crystals were obtained in 0.15 M sodium acetate, 0.1 M Bis-Tris propane (pH 6.5), and 16% PEG 3350 (wt/vol). The scale bar represents 0.3 mm. (C) Diffraction image of an FtsE crystal collected in beamline XALOC at the ALBA synchrotron using a Pilatus 6M detector. Resolution rings are indicated with dashed white lines. (D) Electron-density map (2Fo-Fc map contoured at 1.0 σ) for the 1.36-Å-resolution structure of FtsE in complex with ADP from Streptococcus pneumoniae. The boxed region, comprising the nucleotide-binding pocket, shows a close-up view of the map in which electron densities at 1.36 Å resolution can be appreciated. Download FIG S1, PDF file, 0.5 MB. Copyright © 2020 Alcorlo et al. 2020 Alcorlo et al. This content is distributed under the terms of the Creative Commons Attribution 4.0 International license.

The NBDs present all the functionally critical features belonging to the ABC transporter family (reviewed in references 14 and 44), namely, (i) the Walker A motif (also known as the phosphate-binding loop or P-loop), which binds the α- and β-phosphates of the nucleotide and corresponds to residues 37 to 45 in FtsE (Fig. 2B), (ii) the Walker B motif (residues 160 to 165 in FtsE), which includes the catalytic glutamate (E165), (iii) the A-loop, which corresponds to residues 13 to 17 in FtsE and positions the nucleotide adenine and ribose moieties mainly by an aromatic side chain (Y13 in FtsE), (iv) the switch histidine (H197 in FtsE), which stabilizes the transition-state geometry during ATP hydrolysis, (v) the Q-loop (formed by FtsE residues 86 to 89), named for a conserved glutamine (Q88 in FtsE), which contacts the TMD, (vi) the dimerization or D-loop (residues 168 to 171 in FtsE), which is involved in dimerization and helps couple hydrolysis to transport, and finally, (vii) the α-helical subdomain that contains the signature LSGGQ motif (LSGGE in FtsE, including residues 140 to 144), which pins and orients ATP during hydrolysis and is the hallmark of the ABC transporter superfamily. In summary, FtsE contains all the features and functionally critical regions observed in NBDs of the ABC transporters.

### Structural plasticity in pneumococcal FtsE.

Structural comparison of the four independent FtsE structures reported here (one monomer from the P 1 crystal form and three others, M1, M2, and M3, from the P 2_1_ crystal form associated with chain IDs A, B, and C, respectively) reveals significant differences among them (Fig. S2). These differences are mostly clustered in four regions (Fig. 2C). The first region (R1) is located at the A-loop (affecting residues 12 to 18). The second region (R2) concerns the N-terminal part of α2 (residues 71 to 79), which is slightly displaced among the different structures. The third region (R3, residues 86 to 96) presents a substantial conformational heterogeneity and affects the Q-loop, which connects β6 with α1 and is involved in the interaction with the TMD. In this region, we observe that the side chain of Y90 rotates up to ∼180° depending on the monomer (Fig. S2); as detailed below, this region’s plasticity probably helps accommodate the coupling helix of the TMD. The fourth region (R4, residues 101 to 115) affects the C-terminal part of α3, which is even partially unfolded in monomer 1, resulting in a longer coil loop that protrudes from the monomer structure. The sequence identities corresponding to regions R1 to R4, among the different bacterial species shown in Fig. S3B, are 43%, 78%, 82%, and 66%, respectively. Morphing between the two most structurally different FtsE monomers (monomer from P 1 and monomer 1 from P 2_1_) illustrates the kind of movements observed between lobes I and II of FtsE (Movie S1). The distance and the angle between the two lobes fluctuate up to ∼4 Å and 9° (considering N157 as the hinge between the two lobes), respectively, revealing an unexpected intramolecular breathing motion within the FtsE monomer.

10.1128/mBio.01488-20.2 FIG S2 Structural superposition among the three P 2_1_ monomers and the P 1 monomer. The P 1 monomer is shown in dark blue, and the P 2_1_ monomers are colored in yellow (monomer 1, M1), in green (monomer 2, M2), and in cyan (monomer 3, M3). In the upper panel, for clarity, only one molecule of ADP is shown (gray sticks) at the nucleotide-binding pocket. The lower panel shows a zoomed view of the regions suffering large changes; the change in conformation of Y90 (displayed as capped sticks), which is particularly dramatic in the P 1 monomer, is highlighted. The three P 2_1_ monomers present RMSD of 0.472 Å (M2) and 0.491 Å (M3) for the Cα atoms compared with M1. Superposition of monomer P 1 with monomers in P 2_1_ yields RMSD of 0.862 Å (M1), 0.515 Å (M2), and 0.430 Å (M3) for the Cα atoms. Download FIG S2, PDF file, 1.3 MB. Copyright © 2020 Alcorlo et al. 2020 Alcorlo et al. This content is distributed under the terms of the Creative Commons Attribution 4.0 International license.

10.1128/mBio.01488-20.3 FIG S3 (A) Sequence alignment of FtsE from S. pneumoniae and HisP from *S.* Typhimurium. The percent identity matrix between the two sequences is 31.44% (created with Clustal2.1 [M. A. Larkin, G. Blackshields, N. P. Brown, R. Chenna, et al., Bioinformatics 23:2947–2948, 2007, https://doi.org/10.1093/bioinformatics/btm404]). Secondary structure elements of FtsE are indicated and numbered. Amino acids marked with black or gray boxes indicate sequence identity or similarity, respectively. Sequence gaps are indicated by dashes. Residues corresponding to Walker A/B, A-, D-, and Q-loops, and the signature motif are colored according to the color code shown in Fig. 1C. (B) FtsE sequence alignment among different bacterial species. Identities are boxed in black. Similarities are boxed in gray according to physicochemical properties. (C) Percent identity matrix among the different sequences shown in panel B, calculated with Clustal2.1 (M. A. Larkin, G. Blackshields, N. P. Brown, R. Chenna, et al., Bioinformatics 23:2947–2948, 2007, https://doi.org/10.1093/bioinformatics/btm404). Sequence alignments were produced with T-Coffe (O. Poirot, K. Suhre, C. Abergel, E. O’Toole, et al., Nucleic Acids Res 32:W37–W40, 2004, https://doi.org/10.1093/nar/gkh382) and drawn with ESPript (P. Gouet, E. Courcelle, D. I. Stuart, F. Metoz, Bioinformatics 15:305–308, 1999, https://doi.org/10.1093/bioinformatics/15.4.305). Download FIG S3, PDF file, 0.6 MB. Copyright © 2020 Alcorlo et al. 2020 Alcorlo et al. This content is distributed under the terms of the Creative Commons Attribution 4.0 International license.

10.1128/mBio.01488-20.10 MOVIE S1 Movie showing structural changes between the two most structurally different FtsE monomers (monomer from P 1 and monomer M1 from P 2_1_). Download Movie S1, AVI file, 7.4 MB. Copyright © 2020 Alcorlo et al. 2020 Alcorlo et al. This content is distributed under the terms of the Creative Commons Attribution 4.0 International license.

According to the Pfam database (45), FtsE (25,795 kDa) belongs to the ABC transporter family PF0005. A 3D structural similarity search performed using the DALI server revealed that the closest structure to FtsE in the Protein Database (PDB) is the lipoprotein-releasing system ATP-binding protein LolD from Aquifex aeolicus VF5 (PDB 2PCL; 39% sequence identity and a root mean square deviation (RMSD) of 1.5 Å for 223 superimposed Cα atoms). Comparison with HisP, the periplasmic histidine permease of *Salmonella enterica* serovar Typhimurium (PDB 1B0U [42]) and a well-characterized ABC transporter for this superfamily, indicates 31.4% sequence identity (Fig. S3A) and an RMSD of 2.29 Å for 224 Cα atoms. The main structural differences in FtsE compared with these similar structures (Fig. S4) are concentrated in the ATP-binding loop (A-loop, residues 13 to 18) and the Q-loop (residues 86 to 96), with both regions exhibiting structural plasticity in FtsE (regions R1 and R3 in Fig. 2), the loop 63-72, connecting β5 with α2, which in the case of HisP presents an additional β-hairpin insertion, and finally, the C-terminal region, which in HisP contains a helix-turn-helix motif (HisP residues 236 to 259) but is absent in FtsE. In addition, the C termini of FtsE and HisP are oriented toward the interphase of the two monomer lobes, whereas in LolD, it is oriented in the opposite direction.

10.1128/mBio.01488-20.4 FIG S4 Structural comparison among FtsE homologues. Structural superposition among FtsE (monomer P1, colored in blue), its structurally closest homolog, LolD, from *A. aeolicus* VF5 (PDB 2PCL, colored in orange), and HisP from *S.* Typhimurium (PDB 1B0U, colored in green). The two panels are displayed in two orientations at 90° of each other. These structures are represented in cartoons. Only the ADP molecule from FtsE is shown (in spheres), for clarity reasons. Regions in which differences among these structures are concentrated are indicated. C, C terminus. Download FIG S4, PDF file, 2.6 MB. Copyright © 2020 Alcorlo et al. 2020 Alcorlo et al. This content is distributed under the terms of the Creative Commons Attribution 4.0 International license.

### Nucleotide recognition by FtsE.

**FIG 3:**
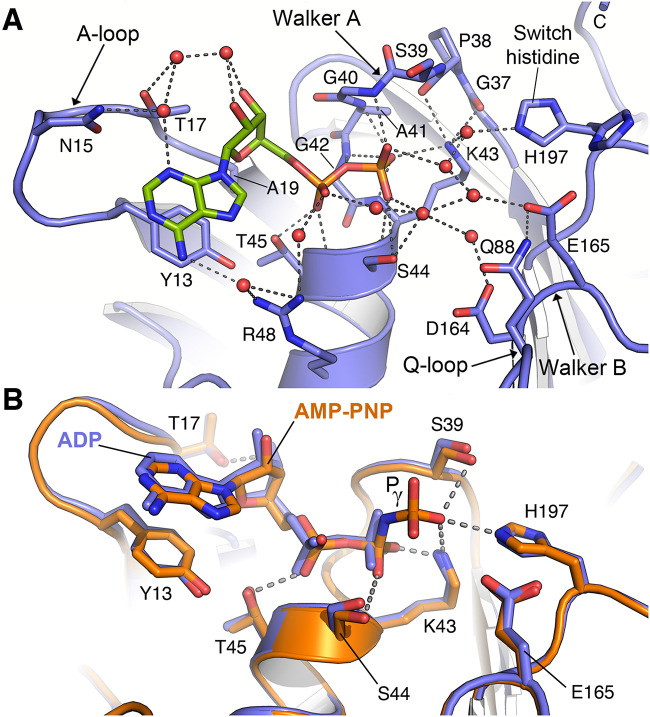
Nucleotide stabilization by FtsE. (A) ADP recognition at the nucleotide-binding pocket of FtsE (P 1 monomer). Relevant residues involved in the ADP interaction are labeled and shown as sticks. Water molecules are represented as red spheres. Dashed lines indicate polar interactions. Atomic interactions were determined using LigPlot (73) with default parameters. (B) Conformational differences between ADP-bound (blue) and AMP-PNP-bound (orange) FtsE monomers (M2 monomers). Residues involved in nucleotide binding, ADP (blue), and AMP-PNP (orange), are depicted as sticks. Phosphate γ is labeled. Dashed lines indicate polar interactions.

FtsE was crystallized with ADP in two different crystal forms (P 1 and P 2_1_) and with AMP-PNP, a nonhydrolyzable analog of ATP, in the space group P 2_1_ (Table 1). The nucleotide-binding site is located in lobe I near the interface between the two lobes and presents the nucleotide sandwiched between the Walker A and B motifs (Fig. 2B). FtsE binds ADP by both van der Waals and polar interactions (Fig. 3A). The Walker A (P-loop) motif wraps over the β-phosphate of ADP forming an extensive hydrogen bond network with main-chain nitrogen atoms of G40 to K43. The position of the K43 side chain, essential for nucleotide binding through interaction with the β-phosphate, is stabilized by H bonds with main-chain oxygen atoms of G37 and P38 (Fig. 3A). The switch histidine (H197) that stabilizes the transition-state geometry in the ADP-bound state interacts with the β-phosphate through two interspersed water molecules. The Walker B motif contains the catalytic glutamate (E65) and Q88, both essential for γ-phosphate hydrolysis in HisP (42) and highly conserved in FtsE proteins from different bacterial species (Fig. S3B). Another conserved residue at the Walker B motif is D164 (Fig. S3B), which in other NBD-ATP complexes, such as HisP (42), interacts with the γ-phosphate through a water molecule that occupies the position of the Mg^++^ in the crystal structures of the Ras-GMPPNP-Mg^++^ (46) and of the F_1_α,β-AMPPNP-Mg^++^ complexes (47). This Asp residue has been suggested to interact with the divalent cation during ATP hydrolysis, by analogy with the process described for GTP hydrolysis in GTPases. The A-loop provides an aromatic side chain (Y13) that packs against the purine ring of adenine involving a π-stacking interaction and positions the base and ribose of the nucleotide. This interaction provides an additional binding free energy for ADP through an extensive hydrophobic interaction that buries 48.4 Å^2^ of surface area (calculated with PDBePISA v1.52) between its aromatic ring and the adenine base of ADP. It is worth mentioning that, in the FtsE monomer, ADP molecules are further stabilized by interactions with a few residues from other monomers in the crystal. In addition, two extra ADP molecules were also found in the P 2_1_ crystal form. They increase crystal contacts (Fig. S5) but are not located in sites with physiological relevance in other ATPases.

10.1128/mBio.01488-20.5 FIG S5 (A) FtsE monomer arrangement in the asymmetric unit of P 2_1_ crystals. The protein is depicted in the ribbon, and each monomer (M1 to M3) is colored differently. ADP is depicted in gray sticks. (B and C) Detailed view of stabilization of the additional ADP (ADP4 and ADP5) molecules found in the P 2_1_ crystal. The protein is represented in a cartoon with relevant residues labeled and shown as capped sticks. ADP4 (B) makes a stacking interaction through its adenine purine rings with the ADP molecule bound to M1. A symmetry-related chain (monomer 1′, colored in gray) contributes to the stabilization of ADP4 through hydrophobic interactions mediated by L78 and F66. ADP5 (C) is found in a cavity formed by three monomers in the crystal and establishing main interactions with M3. The purine adenine ring is sandwiched between Y99 and R112, and the dinucleotide is further stabilized by hydrophobic interaction with M121 and V133 and polar interactions with K117. Download FIG S5, PDF file, 1.8 MB. Copyright © 2020 Alcorlo et al. 2020 Alcorlo et al. This content is distributed under the terms of the Creative Commons Attribution 4.0 International license.

The FtsE–AMP-PNP complex was obtained by soaking with the P 2_1_ FtsE-ADP crystals (Table 1). The ATP analog was found in two out of the three independent monomers of the P 2_1_ crystal. Structural comparison between our ADP and AMP-PNP complexes revealed that there were no relevant structural rearrangements directly associated with AMP-PNP binding, e.g., monomer M2 in complex with ADP and that in complex with AMP-PNP were nearly identical (RMSD of 0.22 Å for 223 Cα atoms) (Fig. 3B). In the presence of AMP-PNP, K43 interacts with both the β- and γ-phosphates. The γ-phosphate is further stabilized by a direct interaction with the switch histidine (H197) and with S39 (Fig. 3B). The catalytic glutamate (E165) is positioned directly adjacent to the γ-phosphate, consistent with a role in ATP hydrolysis. In conclusion, the residues that recognize the ATP molecule are properly oriented, and no relevant changes were required for nucleotide exchange.

### Model for FtsE dimerization.

**FIG 4:**
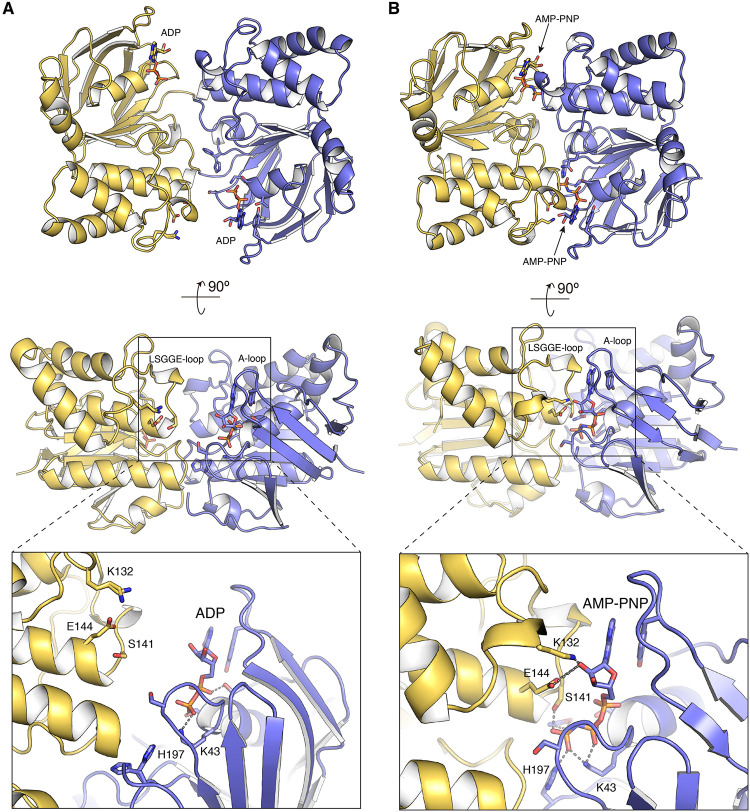
Model for FtsE dimerization in the ADP and ATP states. (A) The FtsE dimer in the ADP-bound conformation. The FtsE-ADP dimer (monomers colored in blue and yellow) as obtained by structural superposition with the protein MacB from A. baumannii (PDB 5GKO [48]). The boxed region at the bottom shows a detailed view of the nucleotide-binding site in the dimer. (B) The FtsE dimer in the ATP-bound conformation. The FtsE–AMP-PNP dimer (monomers colored in blue and yellow) as obtained by structural superposition of FtsE–AMP-PNP with the protein MacB–ATP from A. actinomycetemcomitans (PDB 5LJ6 [49]). The boxed region at the bottom shows a detailed view of the nucleotide-binding site in the ATP dimer. ADP and AMP-PNP molecules and side chains of relevant residues are represented as sticks and labeled. Dashed lines indicate polar interactions of the nucleotide with protein residues.

FtsE performs its catalytic activity *in vivo* as a dimer (13). The availability of structures of ABC transporters in the dimeric state allowed us to build a structural model accounting for the global architecture of the FtsE dimer in the two main, ADP and ATP, physiological states. For the ADP state, we used our FtsE-ADP complex and the crystal structure of tripartite-type ABC transporter MacB from Acinetobacter baumannii in the ADP state (PDB 5GKO [48]) as templates (Fig. S6A). Superposition of the NBD of MacB with the FtsE-ADP complex results in an excellent fit with an RMSD value of 1.0 Å for 174 Cα atoms (Fig. S6B). In this model, FtsE dimerizes in a “head-to-tail” orientation, where each lobe II mutually binds to lobe I of the opposing monomer (Fig. 4A). The interactions between the two subunits in the obtained homodimer model are extensive, burying about 11,493 Å^2^ (calculated with PDBePISA) of accessible surface per subunit. In the FtsE dimer, the ADP nucleotide is stabilized only by a monomer, and thus there is no interaction between the nucleotide and the dimeric partner, in agreement with previous observations for ABC transporters (Fig. 4A).

10.1128/mBio.01488-20.6 FIG S6 (A and B) Structural model of the FtsE dimer in the ADP state. (A) The model was created by superposition of the FtsE-ADP complex (monomer P 1, colored in dark blue) onto the nucleotide-binding domains (NBDs) of the dimer of MacB from A. baumannii (colored in cyan; PDB 5GKO). Superposition presents an RMSD value of 1.0 Å for 174 Cα atoms. ECD, extracellular domain; TMD, transmembrane domain. (Bottom) Detailed view of the superposition of the FtsE-ADP complex onto NBD of MacB. The orientation is similar to Fig. 2B (right). ADP is depicted in capped sticks. (B) The FtsE-ADP dimer shown in a dark blue cartoon displayed in two orientations at 90° of each other. Structural superposition with both NBDs of the dimer of MacB from A. baumannii (cyan cartoon) is also shown. ADP from each FtsE monomer is shown as spheres. (C and D) Structural model of the FtsE dimer in the ATP-bound state. (C) The model was created by superposition of the FtsE–AMP-PNP complex (M1 monomer colored in dark blue) onto the NBDs of the dimer of MacB-ATP from A. actinomycetemcomitans (colored in gray; PDB 5LJ6). ECD, extracellular domain; TMD, transmembrane domain. (Bottom) Detailed view of the boxed area from the top panel comprising one NBD. A composite model in which FtsE was fragmented in three subdomains, lobe I-core (dark blue) including residues 1 to 86, 159 to 164, and 191 to 228, lobe II (yellow) including residues 87 to 158, and α-helix 8 (orange) including residues 165 to 190. Superposition of each region onto the MacB-ATP structure presents RMSD values of 0.8 Å for 125 Cα atoms in the lobe I-core, 0.78 Å for 64 Cα atoms in lobe II, and 0.5 Å for 26 Cα atoms in α-helix 8. The orientation is similar to Fig. 2B (right). ADP is depicted in capped sticks. (D) FtsE dimer model in the ATP-bound state shown in a dark blue cartoon displayed in two orientations at 90° of each other. The structural superposition with both NBDs of the dimer of MacB from A. baumannii (gray cartoon) is also shown. AMP-PNP from each FtsE monomer is shown as spheres. Download FIG S6, PDF file, 0.4 MB. Copyright © 2020 Alcorlo et al. 2020 Alcorlo et al. This content is distributed under the terms of the Creative Commons Attribution 4.0 International license.

To create a model for the FtsE dimer in the ATP state, we used the FtsE–AMP-PNP complex and the crystal structure of the Aggregatibacter actinomycetemcomitans MacB bound to ATP (PDB 5LJ6 [49]) as templates (Fig. S6C). While direct superposition of both structures reveals an overall reasonably good fit (RMSD value of 2.5 Å for 212 Cα atoms), we observed that the MacB-ATP structure presented a more compact structure for the nucleotide-binding domain, with both lobes closer to the central β-sheet II, compared to FtsE. This could potentially arise due to the soaking method used to produce our AMP-PNP complex. We then prepared a composite model in which FtsE was fragmented in three subdomains, lobe I-core (residues 1 to 86, 159 to 164, and 191 to 228), lobe II (residues 87 to 158), and α-helix 8 (residues 165 to 190). Superposition of each region onto the MacB-ATP structure presents excellent RMSD values, namely, 0.8 Å for 125 Cα atoms in lobe I-core, 0.78 Å for 64 Cα atoms in lobe II, and 0.5 Å for 26 Cα atoms in α-helix 8 (Fig. S6C and D), indicating rigid-body movements of these regions around the central β-sheet in the ATP-bound state. In the ATP-bound conformation, there is closer contact between FtsE monomers with the LSGGE-loop of the dimeric partner sandwiching the AMP-PNP bound to the A-loop of the monomer (Fig. 4B). In this model, FtsE residues K132 and E144 of the partner interact with the ribose moiety (Fig. 4B); equivalent residues in MacB (K136 and Q148) also interact with ribose in the ATP complex. It is worth mentioning that in MacB, the γ-phosphate is further stabilized by H bonds to main chain atoms of G147 and A173 of the partner (G142 and N169 in FtsE); in addition, our model predicts the potential implication of the side chain of S141 in the stabilization of the γ-phosphate (Fig. 4B).

In addition, the residues involved in direct protein-protein interactions across the dimer interphase previously described for other NBDs in the dimeric state, such as MalK (PDB 1Q12[50]), are conserved in pneumococcal FtsE. These residues include S38 (S39 in FtsE), which forms strong hydrogen bonds with R141 and D165 (R147 and D171 in FtsE), and H192 (H197 in FtsE), which forms hydrogen bonds and van der Waals interactions with N163, L164, and D165 (N169, L170, and D171 in FtsE).

### Identification of the FtsX region interacting with FtsE in S. pneumoniae.

**FIG 5:**
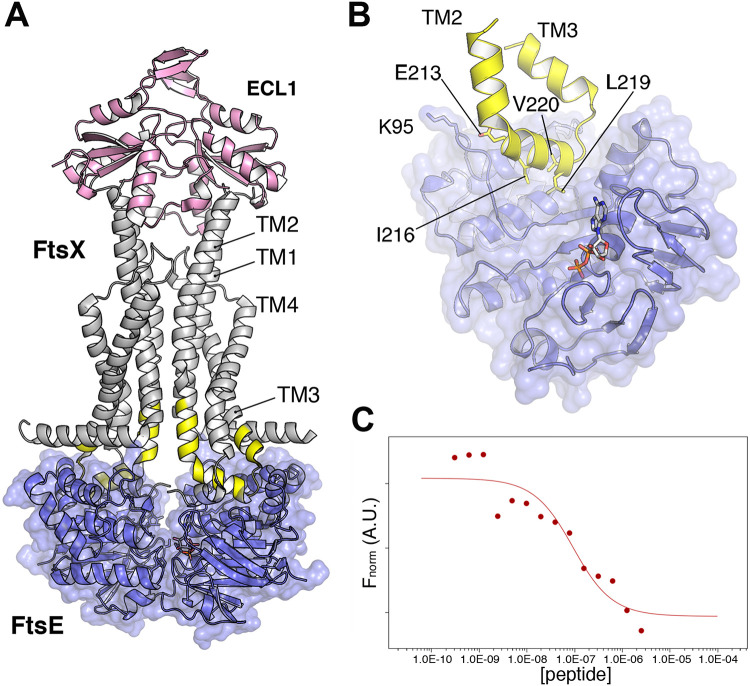
Identification of the FtsX region interacting with FtsE. (A) Full model of the FtsEX complex with the structure of FtsE (this work) colored in blue, the crystal structure of the large extracellular domain of FtsX (ECL1, colored in pink) from S. pneumoniae (PDB code 6HFX [51]), and the transmembrane domain (colored in gray) as observed in MacB from A. Baumannii (PDB code 5GKO [48]). ECL1 from pneumococcal FtsX was structurally superimposed onto the ECD of MacB. The FtsX putative coupling helix is shown as a yellow cartoon. (B) 3D model of the FtsX putative coupling helix (yellow cartoon) obtained with the SWISS-MODEL server (52) using PDB 5NIK (53) as a template. Relevant residues selected for mutagenesis have been labeled and depicted as sticks. FtsE K95, a residue that could interact with FtsX E213, is shown as sticks. The FtsE monomer is shown as a blue cartoon with a semitransparent surface. ADP is depicted as sticks. (C) FtsE interacts with the FtsX peptide ISREIQIMRLVGA. The MST assay was realized with FtsE labeled with dye NT-647. Concentrations on the *x* axis are plotted in M, whereas the the *y* axis shows the normalized fluorescence (MST signal) in arbitrary units. A *K_d_* of ∼82 nM was determined for this interaction.

FtsX is an integral membrane protein with cytoplasmic amino and carboxyl termini, four transmembrane segments, and one large and one small extracellular loop (ECL1 and ECL2, respectively) (Fig. S7A). We recently reported the structural and functional characterization of the large extracellular domain ECL1 from S. pneumoniae (51) connecting the first two transmembrane helices (Fig. 5A and Fig. S7A). In the periplasmic space, ECL1 interacts with the PcsB protein, which harbors a CHAP domain with the endopeptidase activity required for normal cell division. In the cytoplasm, FtsX interacts with FtsE, but the FtsX regions involved in forming the complex in S. pneumoniae are still not known. As described before, FtsE presents a high sequence identity with the nucleotide-binding domains of ABC transporters and also between FtsE proteins from different bacterial species (Fig. S3B and C). Conversely, sequence conservation among FtsX proteins from different bacterial species is rather low (Fig. S7B). This feature probably reflects the fact that FtsX binds PG hydrolases, which vary among different bacterial species (1, 10, 19, 20). Surprisingly, we identified a region of 22 residues with the sequence NTIRITIISRSREIQIMRLVGA (residues 201 to 222 in S. pneumoniae strain R6) predicted as cytosolic and presenting a significant degree of conservation among the different FtsX sequences that we analyzed (Fig. S7B). This region is located between TM2 and TM3 and therefore likely corresponds to the unique cytoplasmic loop present in FtsX. We speculated that the sequence conservation of this region could be due to the interaction with its cytoplasmic partner FtsE. To test this hypothesis, we labeled FtsE with a fluorescent dye and performed microscale thermophoresis (MST) measurements (see Materials and Methods). These experiments show that peptide ISREIQIMRLVGA (the sequence of which is underlined in the 22-residue-long sequence shown above) interacts with FtsE with a dissociation constant (*K_d_*) of ∼82 nM (Fig. 5C). The synthesis of a longer version of the sequence used in this MST analysis failed due to the peptide hydrophobicity. All our attempts to cocrystallize FtsE with this FtsX fragment proved unsuccessful, but a 3D model for this sequence was generated with the SWISS-MODEL server (52) that identified PDB 5NIK as the best template, as it shares 32.26% sequence identity with the query. This structure nicely corresponds to the MacB component of the MacAB-TolC ABC-type tripartite multidrug efflux pump (53), and the region of this structure identified as the template corresponds to an N-terminal helix of the roughly 20 residues that precede TM1 and skirts along the inner leaflet of the cytoplasmic membrane before making an abrupt turn at nearly a right angle into the interior of the lipid bilayer. This N-terminal helix corresponds to the “coupling helix” found in other ABC transporters (54). A groove in the FtsE surface forms the putative contact interface with the coupling helix of the transmembrane domain (TMD). Although the coupling helix is not the only contact between TMDs and NBDs, it is the only architecturally conserved contact among distinct TMD folds and provides the majority of contacts between domains. Based on this 3D model for the FtsX cytoplasmic loop, we identified some potential residues for a mutagenesis study to test whether this FtsX region indeed interacts with FtsE in S. pneumoniae. The selected residues were E213, I216, L219, and V220. Residues I216, L219, and V220 are hydrophobic and would be buried in a cavity present in the FtsE structure that accommodates the FtsX coupling helix (Fig. 5). E213 would protrude outward from the coupling helix establishing polar interactions with residues from FtsE (i.e., K95; Fig. 5B) far away from the FtsE cavity.

10.1128/mBio.01488-20.7 FIG S7 (A) Topology of FtsX from S. pneumoniae. Transmembrane regions are numbered from 1 to 4. ECL, extracellular loop. ECL1 is displayed in a cyan cartoon (PDB 6HFX). (B) Sequence alignment among FtsX proteins belonging to different species. Alignment was carried out using T-Coffe (O. Poirot, K. Suhre, C. Abergel, E. O’Toole, et al., Nucleic Acids Res 32:W37–40, 2004, https://doi.org/10.1093/nar/gkh382) and drawn with ESPript (P. Gouet, E. Courcelle, D. I. Stuart, F. Metoz, Bioinformatics 15:305–308, 1999, https://doi.org/10.1093/bioinformatics/15.4.305). Secondary structure elements of FtsX ECL1 (PDB 6HFX) are indicated and numbered. They are displayed on the top of sequence blocks. Alpha and 310 helices are represented by squiggles labelled α and η, respectively. Strands are represented by arrows. Amino acids marked with red or yellow boxes indicate sequence identity or similarity, respectively. Sequence gaps are indicated by dashes. The FtsX sequence corresponding to the cytoplasmic loop is underlined with a thick black line. Transmembrane helices predicted with the TMHMM server (http://www.cbs.dtu.dk/services/TMHMM/) are indicated with a thick green line running along the top of the sequence alignment and labeled. (C) Model for the FtsE dimer in the ADP-bound form, including the coupling helices from FtsX (gray carton). FtsE monomers are displayed as cartoon putty showing a variable tube representation of the Cα trace in which the thickness of the tube corresponds with higher B-factor values. The FtsE dimer is colored according to the B-factor distribution. Several regions on one of the FtsE monomers are indicated (R1, R2, R3, and R4; see main text for details). TM1 and TM2, FtsX transmembrane region 1 and 2, respectively. ADP is depicted in spheres. A similar result was obtained with the FtsE model in the ATP-bound form (not shown). Download FIG S7, PDF file, 2.2 MB. Copyright © 2020 Alcorlo et al. 2020 Alcorlo et al. This content is distributed under the terms of the Creative Commons Attribution 4.0 International license.

### FtsE interacts with the cytoplasmic region between TM2 and TM3 of FtsX in S. pneumoniae, and this interaction is essential for cell growth and proper morphology.

**FIG 6:**
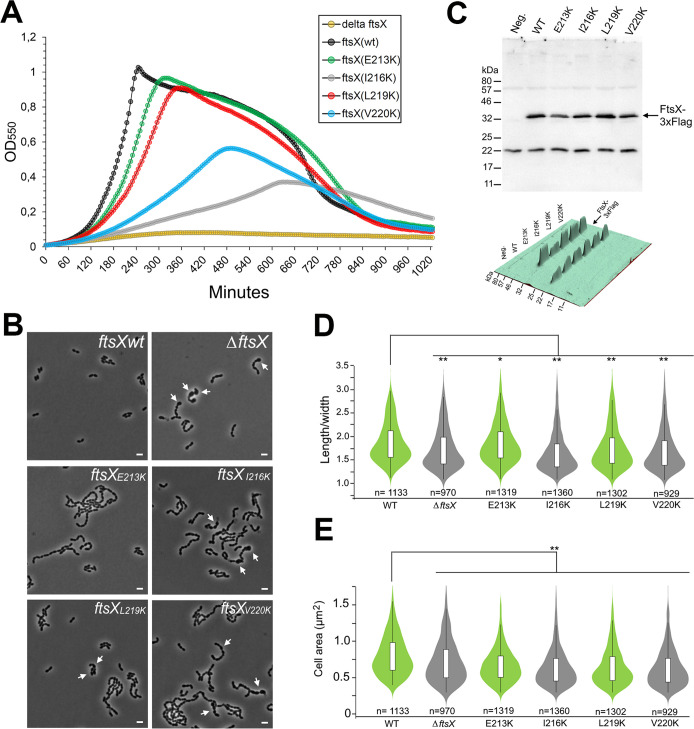
(A) Depletion of ectopic FtsX expression. Representative growth curves of FtsX-depleted mutants are shown (128-fold diluted in well 8 of the microplates; see Materials and Methods). (B) Morphology of pneumococcal strains expressing FtsX^E213K^, FtsX^I216K^, FtsX^L219K^, and FtsX^V220K^ compared to the wild type and a Δ*ftsX* strain. All strains were depleted of an ectopic copy of *ftsX* to test the function of the different *ftsX* versions placed in the native site in the genome. Arrows indicate bloated cells. Scale bars are 2 μm. (C) Stability of point mutant variants of FtsX. Flag-tagged versions of FtsX, FtsX^E213K^, FtsX^I216K^, FtsX^I219K^, and FtsX^V220K^ expressed from the native *ftsX*-locus were detected in whole-cell extracts using immunoblotting. The expected FtsX molecular weight (MW) is 34.2 kDa. The Flag-negative strain RH425 was used as a control for background signals. A 3D visualization of the signal intensities is shown at the bottom. (D and E) Violin plots showing the mean cell/width distribution and cell area (μm^2^) of WT cells and the *ftsX* mutants. (D) The viable mutants are shown in green and the lethal mutations in gray. Comparison of the mean length/width ratio of the WT (1.87 ± 0.42) with Δ*ftsX* (1.74 ± 0.44), *ftsX^E213K^* (1.86 ± 0.43), *ftsX^I216K^* (1.66 ± 0.43), *ftsX^L219K^* (1.76 ± 0.45), and *ftsX^V220K^* (1.69 ± 0.44) cells showed that the ratio of the Δ*ftsX*, *ftsX^I216K^*, *ftsX^L219K^*, and *ftsX^V220K^* mutants were significantly lower than WT cells, with the nonviable mutants I216K and V220K showing the lowest length/width ratios. Mutant E213K did not display a significantly different ratio compared to WT cells. (E) Comparison of the mean cell area of WT cells (0.81 ± 0.28) with the *ftsX* mutants Δ*ftsX* (0.72 ± 0.30), *ftsX^E213K^* (0.68 ± 0.24), *ftsX^I216K^* (0.64 ± 0.26), *ftsX^L219K^* (0.65 ± 0.26), and *ftsX^V220K^* (0.64 ± 0.27). All *ftsX* mutants were significantly smaller than WT cells. The number of cells counted is indicated. *P* values were obtained relative to the WT using one-way analysis of variance (ANOVA). *, *P* > 0.05; **, *P* < 0.001.

Once the potential interacting region between FtsX and FtsE was identified, we then determined the degree to which this interaction interface contributes to pneumococcal viability. To this end, we decided to substitute the above-mentioned residues for lysine (see Materials and Methods). Given the essentiality of the interaction of FtsE and FtsX, the mutation of these residues might have a lethal effect. Thus, we used a strain containing an ectopic copy of *ftsX* under the control of the inducible P*_comR_* promoter (see Materials and Methods) that allows the depletion of ectopic FtsX expression by using the ComRS gene expression/depletion system. Subsequently, mutants were inspected for growth or morphology defects. By growing the cells without ComS inducer in a 2-fold dilution series, the level of intracellular ComS is gradually reduced through cell division and metabolism. To test the functionality of different FtsX mutants, ectopic FtsX was depleted in the following genetic backgrounds: Δ*ftsX*, *ftsX_wt_*, *ftsX^E213K^*, *ftsX^I216K^*, *ftsX^L219K^*, and *ftsX^V220K^*. All mutants tested presented various degrees of phenotypic defects such as reduced growth and chaining (Fig. 6A and B). Comparison of their cell shape distributions (length/width) showed that the most severely affected mutants, I216K and V220K, also resemble the *ftsX*-depleted cells in being less elongated than wild-type (WT) cells and having a significant number of bloated cells (Fig. 6B and D). The L219K mutant also displayed shorter cells and bloated cells, whereas the E213K mutant did not. However, when the mean cell area (μm^2^) was compared, all FtsX mutants were in general smaller than the WT (Fig. 6E), despite the contribution of several bloated cells seen for the nonviable mutants. We also constructed Flag-tagged versions of the point mutated FtsX proteins to check whether their expression levels and stability were similar to WT FtsX when expressed from the native promoter (Fig. 6C). This analysis revealed that all mutants were expressed at nearly WT FtsX levels, indicating that the observed differences were not due to misexpression of FtsX. However, the levels of E213K appeared to be somewhat reduced compared to the others. We do not believe this is a critical issue, since this mutant was viable. Although it displayed chaining and a slight growth reduction, the cell’s length/width ratios were not significantly different from WT cells. The I216K and V220K mutants, which were lethal, are expressed at WT levels, confirming that it is not the lack of FtsX protein that caused this phenotype, but the loss of interaction with FtsE. The E213K mutant has the mildest phenotype, probably because this residue perturbs outside the hydrophobic cavity present in FtsE.

Taken together, these results reveal that the cytoplasmic region of FtsX that links TM2 and TM3 is critical for FtsX function *in vivo* and confirm the functional importance of the physical interaction of FtsX and FtsE.

## DISCUSSION

FtsEX is a member of a subclass of ABC transporters believed to transmit a conformational change to accomplish work in the periplasm. Two ABC transporters, called MacB and LolCDE, are closely related to FtsEX, as they have the same transmembrane topology and an extracellular domain (ECD) located between the first and second membrane-spanning helices. Both these proteins have four TM segments and assemble into ABC transporters as dimers with a total of eight TM segments (55–57). LolC and LolD work together with the cytoplasmic ATPase LolE to transfer lipoproteins from the outer surface of the cytoplasmic membrane to the periplasmic chaperone LolA (58, 59). MacB helps to secrete heat-stable enterotoxin II from the periplasm across the outer membrane (60). In both cases, their activity does not form a substrate channel to move a substrate across a membrane; instead, the TM segments serve to transmit ATP-driven conformational changes in a process known as mechanotransmission (49). The FtsEX complex is expected to behave in this same manner, coupling cytoplasmic ATP hydrolysis with extracellular conformational changes that drive the activation of the PG hydrolytic activity of PcsB during bacterial division (Fig. 1).

**FIG 7:**
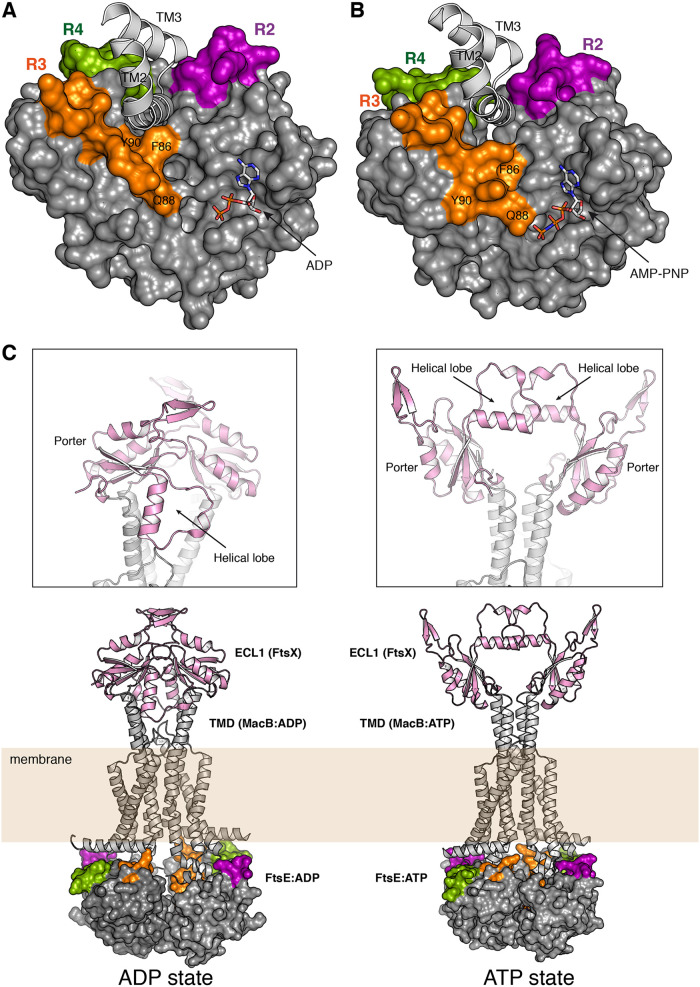
(A) Model of the FtsE-ADP complex with the FtsX coupling helix. The molecular surface of the FtsE-ADP complex (monomer P 1) is colored in gray with regions R2 (residues 71 to 79), R3 (residues 86 to 96), and R4 (residues 101 to 115) colored in purple, orange, and green, respectively. TM helices from MacB from A. baumannii (PDB 5GKO [48]) are represented as light gray ribbons. (B) Model of the FtsE-ATP complex with the FtsX coupling helix. The molecular surface of the composite model of the FtsE–AMP-PNP complex was obtained by superimposition onto the A. actinomycetemcomitans MacB bound to ATP (PDB 5LJ6 [49]). Regions R2 (residues 71 to 79), R3 (residues 86 to 96), and R4 (residues 101 to 115) are highlighted in purple, orange, and green, respectively. TM helices from MacB:ATP are represented as light gray ribbons. ADP and AMP-PNP molecules are represented as sticks. Some relevant residues (see main text for details) are labeled. (C) Full model of the pneumococcal FtsEX complex in the ADP and ATP states. The TM helices correspond to the MacB complex in the ADP or ATP states (PDB codes 5GKO and 5LJ6, respectively). The position of the pneumococcal ECL1 of FtsX (PDB code 6HE6) was obtained by direct superimposition of the Porter subdomain of FtsEx onto the Porter subdomain of MacB. Details of the arrangement of the helical lobes of FtsX in the ADP and ATP states are shown in the boxed areas.

The disposition of similar NBD structures in complex with their corresponding TMDs (48, 49) allowed us to generate a model for the pneumococcal FtsE dimer in both the ADP- and ATP-bound conformations in complex with the FtsX coupling helix. Interestingly, three out of the four FtsE regions exhibiting conformational plasticity (regions R2, R3, and R4) shape the cavity in which the FtsX coupling helix would be located (Fig. 7A and B). In agreement with its dynamic behavior, these FtsE regions present above-average temperature factors (Fig. S7C) that, together with their close proximity with the TMD, favor the transmission of a conformational change to FtsX. The bottom of the cavity is mainly composed of hydrophobic residues, among them F87 (Fig. 7A). The relevance of these hydrophobic interactions agrees with our observation that FtsX mutants affecting hydrophobic residues at the cytoplasmic loop (I216K, L219, and V220K) have a dramatic phenotype affecting cell growth and division.

Other conclusions can be drawn from our structures; in particular, we have observed that while the relaxed structure of the FtsE-ADP complex nicely fits the equivalent complex in the MacB dimer; the monomer of the FtsE–AMP-PNP complex requires rigid-body adjustment of some regions around the central β-sheet in order to get a perfect fit with the more compact structure of the MacB-ATP dimer. Considering the high sequence identity among the NBD domains of ABC transporters, this result indicates that protein-protein interactions in the dimer are required for full recognition of the ATP nucleotide, just as we predicted with our ATP-bound FtsE dimer model (Fig. 4B), and to reach the final 3D structure *in vivo*. The observed plasticity in FtsE would favor such protein-protein mediated changes as also occur in our crystal structures.

The disposition and conformation of FtsE R2-R4 regions is different in ADP versus ATP-bound states (Fig. 7A and B), thus altering the global shape of the FtsX-binding cavity. Structural changes observed in the backbone or in the side chain conformation of some residues (such as Y90) in these regions would correlate with the changes between ADP and ATP conformations in FtsE. For instance, in our FtsE–AMP-PNP complex, the γ-phosphate is far from the Q88 residue of the Q-loop (>6 Å); however, our composite model for the same structure in the dimer indicates that Q88 would directly recognize the γ-phosphate (Fig. 7B), as also occurs in the structure of the MacB-ATP dimer (49). In conclusion, FtsE regions R2 to R4 would play a relevant role in the transmission from the cytosol through the membrane in order to regulate lytic activity in the periplasm.

To further explore which FtsX region is recognized by FtsE in S. pneumoniae, we performed an *in vivo* mutagenesis study. Our results clearly pinpoint the FtsX cytoplasmic loop (residues 201 to 222) linking TM2 and TM3 as an FtsE binding site. Our results are in agreement with a recent study in which the authors show that mutations affecting this FtsX region in E. coli markedly reduced the ability of FtsEX to complement a Δ*ftsEX* mutant (12).

How mechanotransmission enables FtsEX to affect PcsB is a central question that still remains unanswered; i.e., how do the ATP- versus ADP-bound forms of FtsE in the cytosol affect ECL1 at the periplasm, which then affects PcsB? While the final answer will likely come from the 3D structures of the binary and ternary complexes (FtsEX and FtsEX-PcsB), the present structural information can provide some insights into this matter. The structure of pneumococcal ECL1 of FtsX (51) presents a helical lobe and the so-called Porter subdomain, which is remarkably similar to that of E. coli MacB (1, 49). The Porter subdomain is present in all members of the type VII ABC superfamily and is likely an intrinsic part of the transmission apparatus (18). Structural superposition of the Porter subdomains in the pneumococcal ECL1 and in MacB, in both the ATP and ADP states, has allowed us to generate a 3D model accounting for the arrangement of the FtsX ECL1 between the two states (Fig. 7C). Interestingly, depending on the ADP or ATP state, the helical lobe of ECL1 changes its orientation completely. In the ATP state, the helical lobes from each ECL1 are very close and oriented face to face, while in the ADP state, the helical lobes of each ECL1 face in opposite directions (Fig. 7C). We previously reported that the ECL1 helical lobe mediates a physical interaction with PcsB (47). Thus, this difference between the ATP and ADP states would dramatically alter the FtsEX-PcsB interaction. This deduction could be applied to other bacteria, such as E. coli, where it was shown that this same FtsX lobe (referred to as lobe X in Gram-negative bacteria [6]) is required for binding to EnvC (10). In our model, the secondary structural elements that make up ECL1 are not altered, and just a rotation of ECL1 dictated by the scissor-like action of the transmembrane domains driven by the dimerization of the FtsE would be needed to provide the driving force necessary to regulate lytic activity in the pneumococcal division.

Considering the conservation among bacterial species and the relevance of this system in cell viability, a deep understanding of cell division regulation by FtsEX could serve as an important base to design new classes of antibiotics.

## MATERIALS AND METHODS

### Cloning, expression, and purification of protein FtsE.

We fused FtsE at its N terminus to a 6×His-tag followed by a tobacco etch virus (TEV) protease cleavage site in vector pETDuet-1 (Novagen) that allows tunable expression of the fusion protein from a T7 promoter induced by isopropyl-β-d-1-thiogalactopyranoside (IPTG). Briefly, the DNA fragment corresponding to *ftsE*, flanked by EcoRI and HindIII restriction sites, was amplified by PCR from genomic DNA of S. pneumoniae strain R6 (primers Ftse1 and Ftse2; Table S2), digested with restriction enzymes EcoRI and HindIII, and cloned into equivalent sites of vector pETDuet-1 at the multicloning site 1. Escherichia coli DH5α cells were transformed with this ligation mixture, and ampicillin-resistant transformants were selected. The resulting construct was verified by Sanger sequencing and used to transform E. coli Lemo21. This strain (BL21DE3/pETDuet-1-*6xhis-TEV-ftsE*) was grown until an optical density at 600 nm (OD_600_) of 0.5 was reached, and His-FtsE expression was induced with 0.3 mM IPTG (Promega) for 3 h at 28°C. Cell pellets were resuspended in buffer E (50 mM Tris-HCl [pH 7.5], 500 mM NaCl, 20 mM imidazole, 2 mM ATP, and 10% glycerol) containing 1 mmol/liter phenylmethylsulfonyl fluoride (PMSF) and DNase I (Roche Diagnostics Corp., Indiana, USA) to 5 μg/ml. Cell suspensions were sonicated on ice for 5 min at 35% amplitude. Cell lysates were centrifuged at 10,000 × *g* at 4°C for 40 min. Supernates were loaded by gravity onto a Ni^+2^-charged column (HisTrapHP, GE Healthcare) equilibrated with buffer E. The column was washed with 20 mM imidazole in buffer E until protein could not be detected using the Bradford protein assay, and His-FtsE was eluted by gradually increasing the concentration of imidazole from 20 to 500 mM. Eluted proteins were collected in 1-ml fractions and examined using SDS-PAGE. Fractions with the highest purity were pooled and dialyzed overnight against 25 mM Tris-HCl (pH 7.5), 2 mM DTT, and 100 mM NaCl at 4°C. The His-tag of the dialyzed protein was cleaved off by digestion with His-tagged TEV protease at 30°C for 3 h. Undigested proteins, free His-tag, and His-tagged TEV were separated from digested FtsE by performing a second immobilized metal affinity chromatography step with a Ni^+2^-charged column. The digested FtsE protein was collected in the flowthrough and concentrated up to 8 mg/ml using a Millipore ultraconcentrator (10 kDa cutoff). The concentration of the purified FtsE was determined by absorbance at 280 nm using an extinction coefficient of ε = 15,930 M^−1 ^cm^−1^ calculated by the ExPASy ProtParam program (61).

### FtsE crystallization.

Crystallization screenings were performed using high-throughput techniques in a NanoDrop robot and Innovadyne SD-2 microplates (Innovadyne Technologies, Inc.) with screening PACT Suite and JCSG Suite (Qiagen), JBScreen Classic 1 to 4 and 6 (Jena Bioscience), and Crystal Screen, Crystal Screen 2, and Index HT (Hampton Research). The conditions that produced crystals were optimized with a sitting-drop vapor-diffusion method at 291 K by mixing 1 μl of protein solution and 1 μl of precipitant solution, equilibrated against 150 μl of precipitant solution in the reservoir chamber. The best crystals were obtained in a crystallization condition containing 0.15 M sodium acetate, 0.1 M Bis-Tris propane (pH 6.5), and 16% (wt/vol) PEG3350 (Fig. S1B). Protein concentration was assayed at the concentration of 8 mg/ml.

### Soaking experiments with AMPPNP.

For soaking trials, AMPPNP was dissolved in water, and the pH was adjusted to 7.0 by addition of NaOH. Next, AMPPNP was incubated with native protein crystals at a final concentration of 5 mM using the crystallization conditions described above. Soaking experiments were incubated overnight at 291 K.

### X-ray data collection, phasing, and model refinement.

Diffraction data were collected in beamlines ID23-1 at the European Synchrotron Radiation Facility (ESRF) and XALOC at the ALBA synchrotron using the Pilatus 6M detector. Crystals diffracted up to 1.36 to 2.40 Å resolution and belonged to the P 1 or P 2_1_ space groups with the unit cell parameters *a *= 33.39 Å, *b *= 36.71 Å, *c *= 41.11 Å, α = 105.27°, γ = 95.69°, and β = 99.69° and *a *= 33.11 Å, *b *= 118.79 Å, *c *= 82.48 Å, α = γ = 90°, and β = 97.51°, respectively. The collected data sets were processed with XDS (62) and Aimless (63). In the P 1 crystals, one monomer was found in the asymmetric unit, yielding a Matthews coefficient (64) of 1.86 Å^3^/Da and a solvent content of 33.83%. In the P 2_1_ crystals, three monomers were found in the asymmetric unit, yielding a Matthews coefficient of 2.08 Å^3^/Da and a solvent content of 40.78%. For crystals belonging to space group P 1, the structure determination was performed with *de novo* phasing with ARCIMBOLDO (41). We used a search including 7 copies of a helix containing 6, 8, 10, 12, 13, 15, and 16 residues, respectively, assuming an RMSD from the target of 0.2 Å. For crystals belonging to space group P 2_1_, the structure determination was performed with the molecular replacement method.

In both cases, the model was manually completed using Coot (65) followed by refinement using PHENIX (66). A summary of the refinement statistics is given in Table 1.

### Microscale thermophoresis experiments.

We ordered the synthetic sequence NTIRITIISREIQIMRLVGAKNSYIRG (JPT Peptide Technologies GmbH) for assaying the interaction with FtsE, but this peptide is extremely hydrophobic and could not been synthetized. Instead, we used a shorter version, ISREIQIMRLVGA, that could be successfully synthesized. For performing MST experiments, fluorescent label dye NT-647 was covalently attached to the protein. The concentration of labeled FtsE was kept constant (10 μM), while the concentration of nonlabeled peptide was varied between 10 μM and 0.3 nM. The assay was performed in a buffer comprising 25 mM Tris-HCl (pH 7.5), 150 mM NaCl, and 5 mM ATP. After 30 min of incubation at room temperature (RT), the samples were loaded into Monolith NT.115 premium treated capillaries, and the MST analysis was performed using a Monolith NT.115 instrument. Unfortunately, we could not obtain any complex either by soaking or by cocrystallization with the indicated peptide.

### Cultivation and transformation of S. pneumoniae.

S. pneumoniae was grown in liquid C medium (67) or on Todd-Hewitt (BD Difco) agar plates at 37°C. Plates were incubated in an anaerobic chamber made by including AnaeroGen bags from Oxoid in a sealed container. For selection of transformants, kanamycin or streptomycin was added to the growth medium to a final concentration of 400 or 200 μg/ml, respectively. Genetic transformation of S. pneumoniae was performed by natural competence as previously described by Straume et al. (68).

### Construction of S. pneumoniae mutants.

Construction of gene knockout cassettes and amplicons used to introduce genetic mutations in S. pneumoniae was done by using overlap extension PCR as previously described by Johnsborg et al. (69). The primers used are listed in Table S2. Amplicons were flanked 5′ and 3′ with ∼1,000 bp homologous with sequences flanking the target site in the S. pneumoniae genome, allowing double crossover via homologous recombination after transformation. Gene knockouts were confirmed by PCR, and mutations were verified by Sanger sequencing. The strains used in this work are listed in Table S1.

10.1128/mBio.01488-20.8 TABLE S1 S. pneumoniae R6 strains used in the present study. Download Table S1, PDF file, 0.1 MB. Copyright © 2020 Alcorlo et al. 2020 Alcorlo et al. This content is distributed under the terms of the Creative Commons Attribution 4.0 International license.

10.1128/mBio.01488-20.9 TABLE S2 Primers used in the present study. Download Table S2, PDF file, 0.1 MB. Copyright © 2020 Alcorlo et al. 2020 Alcorlo et al. This content is distributed under the terms of the Creative Commons Attribution 4.0 International license.

### Depletion of FtsX and microscopy.

To deplete ectopic expression of FtsX in S. pneumoniae, we used the ComRS gene expression/depletion system (70). For the growth experiment, strains ds312, ds314, ds751, ds753, ds754, and ds761 were grown to an OD_550_ of 0.2 in 5 ml C medium containing 0.5 μM ComS inducer. Then the cells were collected at 4,000 × *g* and washed once in 5 ml C medium without ComS. To start *ftsX* depletion, the cells were diluted to an OD_550_ of 0.05 in C medium without ComS. The cultures were further diluted 2-fold in ComS-free C medium in a series of 12 tubes, and 300 μl from each dilution was transferred to a 96-well microtiter plate. The plate was incubated at 37°C, and the OD_550_ was measured automatically every fifth minute in a Synergy H1 hybrid reader (BioTek). For microscopic examinations of cells depleted of ectopically expressed FtsX, the strains ds312, ds314, ds751, ds753, ds754, and ds761 were pregrown and washed as described above. Then the cultures were further diluted 128 times (corresponding to well 8 in the microtiter plate experiment) in ComS-free C medium to a final volume of 10 ml. At an OD_550_ of 0.2 or when the growth stopped at a lower density due to depletion of ectopic FtsX, cells were immobilized on a microscopy slide using 1.2% agarose. Images of the cells were taken using a Zeiss AxioObserver microscope with ZEN Blue software and an ORCA-Flash 4.0 V2 digital complementary metal oxide semiconductor (CMOS) camera (Hamamatsu Photonics) with a 1,003 phase-contrast objective. Images were prepared in ImageJ, and cell detection for morphogenesis analyses was done using the MicrobeJ plugin (71).

### Immunodetection of Flag-tagged FtsX.

Flag-tagged versions of FtsX expressed in the native *ftsX* locus of strains ds768, ds769, ds770, ds771, and ds772 were detected by immunoblotting. The cells were grown in the presence of 0.05 μM ComS inducer to drive expression of ectopic FtsX. Cells from 10-ml cultures with an OD_550_ of 0.2 were collected at 4,000 × *g* and resuspended in 200 μl SDS sample buffer. The samples were heated at 95°C for 10 min before proteins were separated by SDS-PAGE using a 12% separation gel. The proteins were then electroblotted onto a polyvinylidene difluoride (PVDF) membrane (Bio-Rad), and Flag-tagged FtsX proteins were detected by using anti-FLAG antibody (Sigma) as previously described by Stamsås et al. (72) except that both the primary and secondary anti-rabbit-horseradish peroxidase (HRP) antibodies (Sigma) were diluted 1:4,000 in Tris-buffered saline with Tween 20 (TBS-T).

### Data availability.

Crystallographic data were submitted to the PDB under codes 6Z4W, 6Z63, and 6Z67.
