# Supplementary material for: Structural Characterization of the Essential Cell Division Protein FtsE and Its Interaction with FtsX in Streptococcus pneumoniae
Source: mBio. 2020 Sep 1;11(5):e01488-20. doi: 10.1128/mBio.01488-20 (PMC7468199; doi:10.1128/mBio.01488-20)
Supplement: FIG S1 [file mBio.01488-20-sf001.pdf]

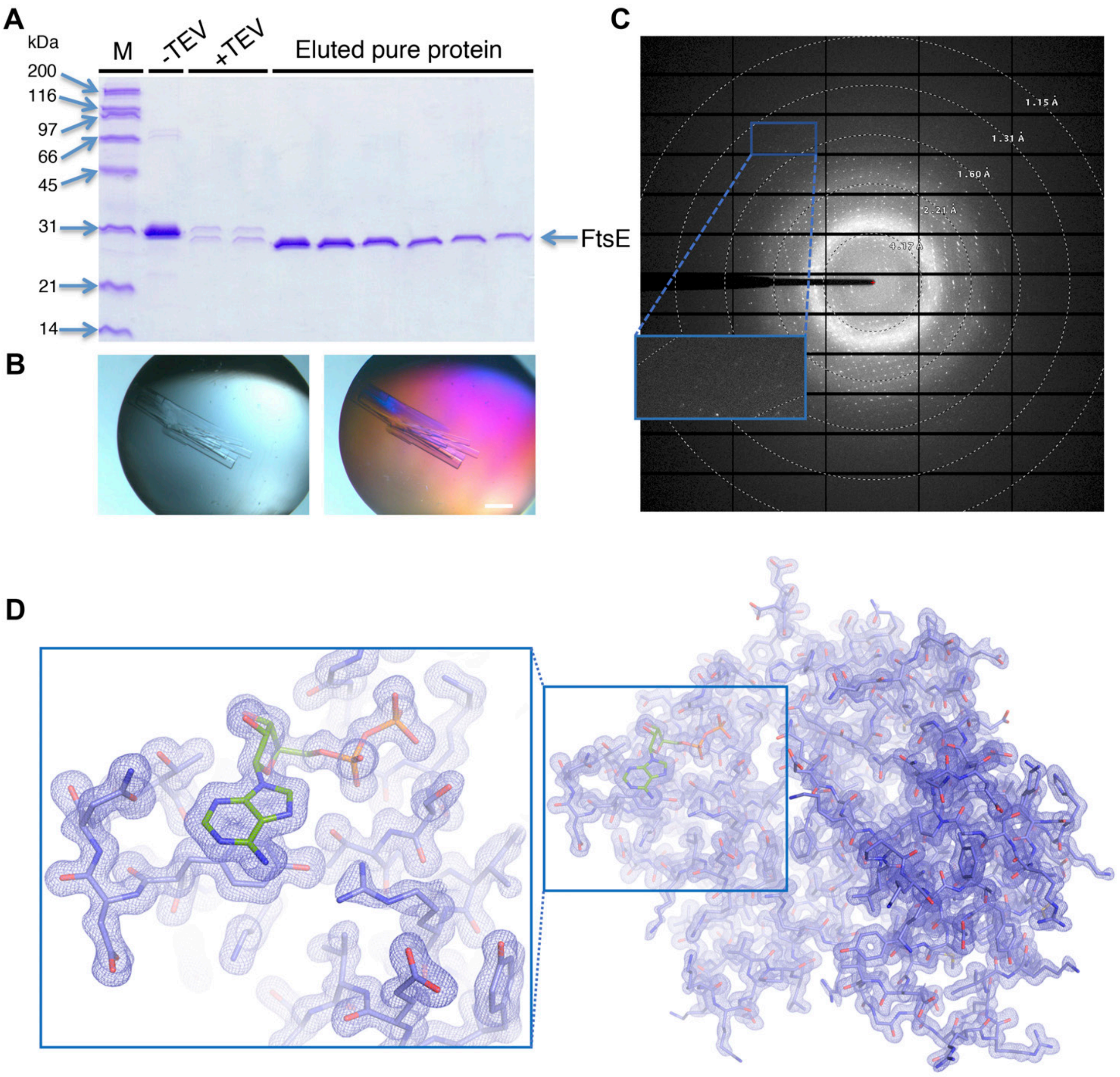

Fig. S1. (**A**) SDS-PAGE (12%) analysis of the eluted pure FtsE protein after His-tag removal with TEV protease. Molecular weight-markers (kDa) are indicated for the left lane. (**B**) FtsE crystals belonging to space group P 2_1 diffracted up to 1.57 Å resolution. Crystals were obtained in 0.15 M sodium acetate, 0.1 M Bis-Tris propane pH 6.5 and 16% PEG 3350 (w/v). Scale bar represents 0.3 mm. (**C**) Diffraction image of a FtsE crystal collected in beamline XALOC at the ALBA synchrotron using a Pilatus 6M detector. Resolution rings are indicated with dashed white lines. (**D**) Electron-density map (2Fo-Fc map contoured at 1.0 σ) for the 1.36 Å resolution structure of FtsE in complex with ADP from *Streptococcus pneumoniae*. The boxed region, comprising the nucleotide binding pocket, shows a close-up view of the map in which electron densities at 1.36 Å resolution can be appreciated.
