# Supplementary material for: Structural Characterization of the Essential Cell Division Protein FtsE and Its Interaction with FtsX in Streptococcus pneumoniae
Source: mBio. 2020 Sep 1;11(5):e01488-20. doi: 10.1128/mBio.01488-20 (PMC7468199; doi:10.1128/mBio.01488-20)
Supplement: FIG S2 [file mBio.01488-20-sf002.pdf]

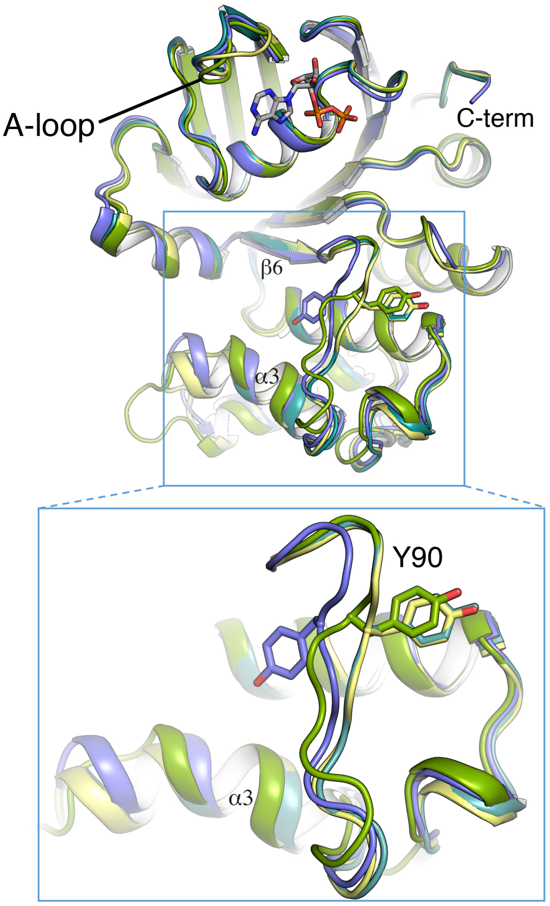

Fig. S2. Structural superposition among the three P 2_1 monomers and the P 1 monomer. The P 1 monomer is shown in dark blue and the P2_1 monomers are colored in yellow (monomer 1, M1), in green (monomer 2, M2) and in cyan (monomer 3, M3). In the upper panel, for clarity only one single molecule of ADP is shown (gray sticks) at the nucleotide-binding pocket. Lower panel shows a zoomed view of the regions suffering large changes; the change in conformation of Y90 (displayed in capped sticks), which is particularly dramatic in the P 1 monomer, is highlighted. The three P 2_1 monomers present *rmsd* of 0.472 Å (M2) and 0.491 Å (M3) for the Cα atoms when compared with M1. Superposition of monomer P 1 with monomers in P21 yields *rmsd* of 0.862 Å (M1), 0.515 Å (M2) and 0.430 Å (M3) for the Cα atoms.
