# Supplementary material for: Structural Characterization of the Essential Cell Division Protein FtsE and Its Interaction with FtsX in Streptococcus pneumoniae
Source: mBio. 2020 Sep 1;11(5):e01488-20. doi: 10.1128/mBio.01488-20 (PMC7468199; doi:10.1128/mBio.01488-20)
Supplement: FIG S3 [file mBio.01488-20-sf003.pdf]

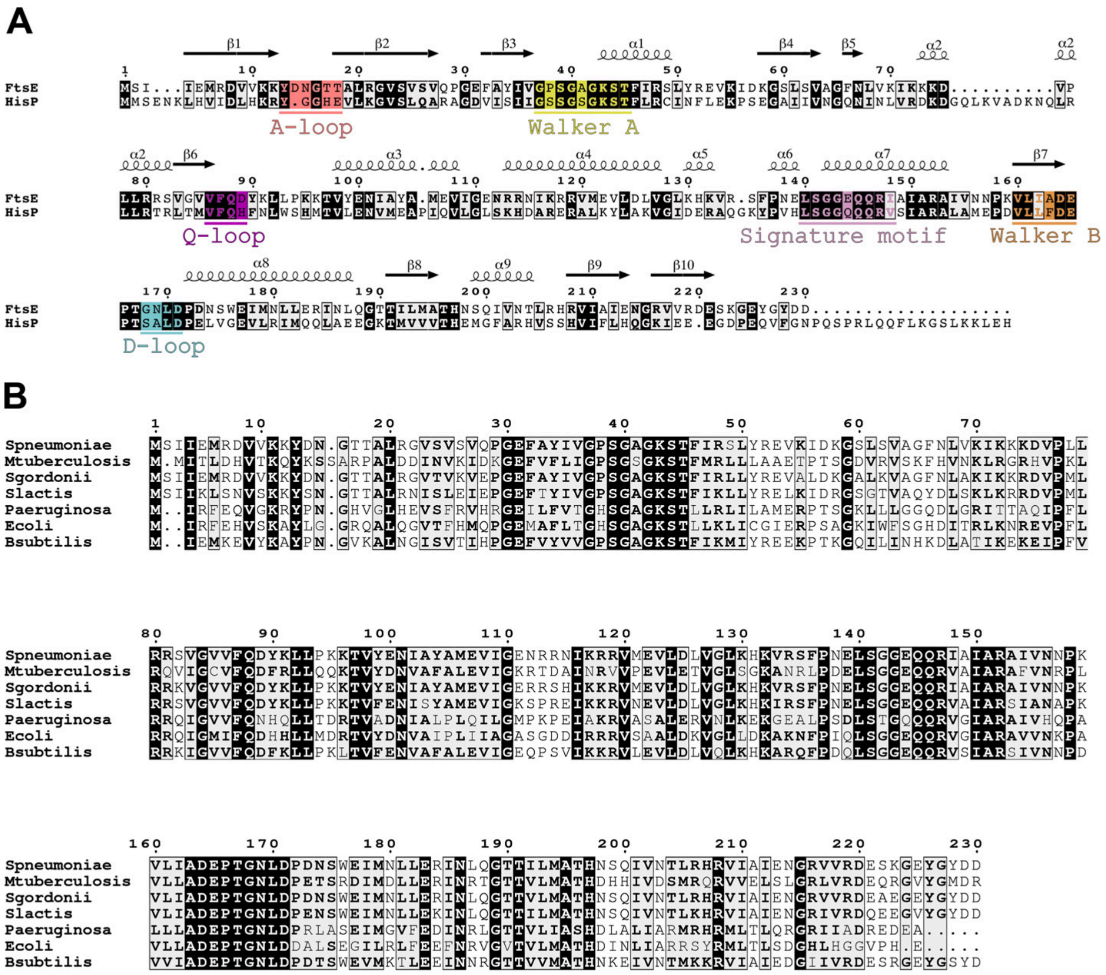

C

Percent Identity Matrix

	1	2	3	4	5	6	7
1: *Paeruginosa*	100.00	54.05	43.50	44.39	40.81	42.15	43.05
2: *Ecoli*	54.05	100.00	47.75	44.59	41.44	42.34	43.24
3: *Mtuberculosis*	43.50	47.75	100.00	50.88	51.53	51.97	53.71
4: *Bsubtilis*	44.39	44.59	50.88	100.00	63.16	64.91	65.35
5: *Slactis*	40.81	41.44	51.53	63.16	100.00	79.13	78.70
6: *Spneumoniae*	42.15	42.34	51.97	64.91	79.13	100.00	91.74
7: *Sgordonii*	43.05	43.24	53.71	65.35	78.70	91.74	100.00

Fig. S3. (**A**) Sequence alignment of FtsE from *S. pneumoniae* and HisP from *S. typhimurium*. The percent Identity Matrix between the two sequences is 31.44 % [created by Clustal2.1 (1)]. Secondary structure elements of FtsE are indicated and numbered. Amino acids marked with black or gray boxes indicate sequence identity or similarity, respectively. Sequence gaps are indicated by *dashes*. Residues corresponding to Walker A/B, A-, D- and Q-loops, and the signature motif have been colored according to the color code shown in Fig. 1C. (**B**) FtsE sequence alignment among different bacterial species. Identities are boxed in black. Similarities are boxed in gray according to physico-chemical properties. (**C**) Percent Identity Matrix among the different sequences shown in B, calculated with Clustal2.1 (1). Sequence alignments were produced by T-Coffe (3) and drawn with ESPript (2).

References:

1. Madeira F, Park YM, Lee J, Buso N, Gur T, Madhusoodanan N, Basutkar P, Tivey ARN, Potter

SC, Finn RD, Lopez R. 2019. The EMBL-EBI search and sequence analysis tools APIs in 2019. Nucleic Acids Res2019/04/13. 47:W636–W641.
2. Gouet P, Courcelle E, Stuart DI, Metoz F. 1999. ESPript: analysis of multiple sequence alignments in PostScript. Bioinformatics1999/05/13. 15:305–308.
3. Poirot O, Suhre K, Abergel C, O'Toole E, Notredame C. 2004. 3DCoffee@igs: a web server for combining sequences and structures into a multiple sequence alignment. Nucleic Acids Res2004/06/25. 32:W37-40.
