# Supplementary material for: Structural Characterization of the Essential Cell Division Protein FtsE and Its Interaction with FtsX in Streptococcus pneumoniae
Source: mBio. 2020 Sep 1;11(5):e01488-20. doi: 10.1128/mBio.01488-20 (PMC7468199; doi:10.1128/mBio.01488-20)
Supplement: FIG S4 [file mBio.01488-20-sf004.pdf]

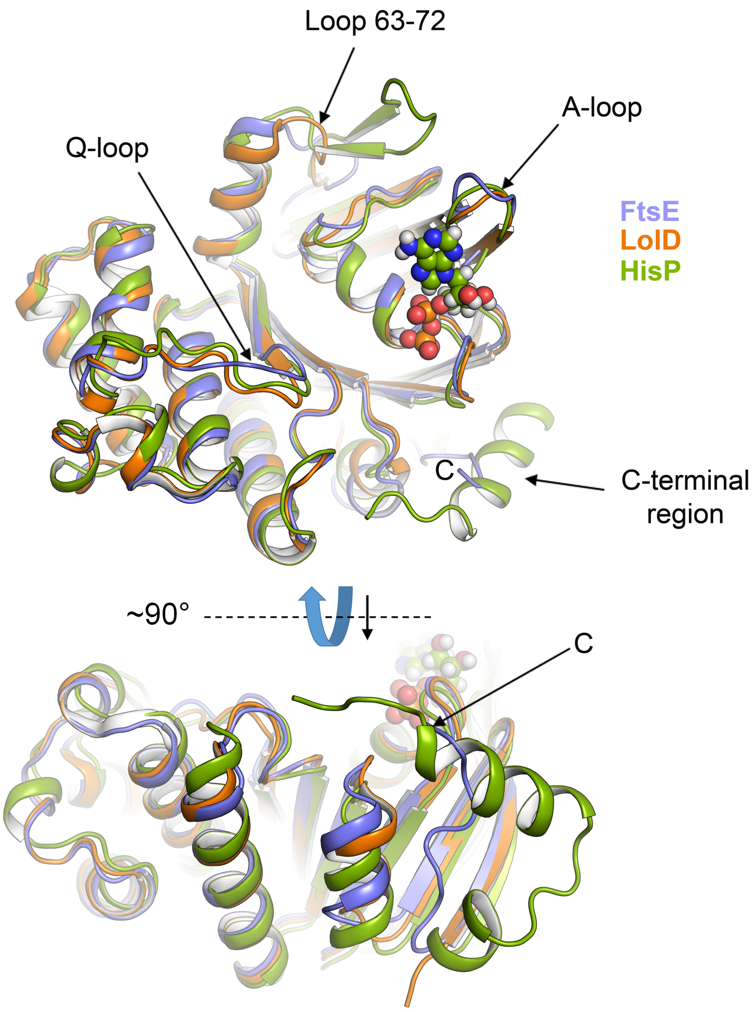

Fig. S4. Structural comparison among FtsE homologues. Structural superposition among FtsE (monomer P1, colored in blue), its structurally closest homolog LolD from *A. aeolicus* VF5 (PDB 2PCL, colored in orange) and HisP from *S. typhimurium* (PDB 1B0U, colored in green). The two panels are displayed in two orientations at 90° of each other. These structures are represented in cartoon. Only the ADP molecule from FtsE is shown (in spheres) for clarity reasons. Regions in which differences among these structures are concentrated are indicated. C, C-terminus.
