# Supplementary material for: Structural Characterization of the Essential Cell Division Protein FtsE and Its Interaction with FtsX in Streptococcus pneumoniae
Source: mBio. 2020 Sep 1;11(5):e01488-20. doi: 10.1128/mBio.01488-20 (PMC7468199; doi:10.1128/mBio.01488-20)
Supplement: FIG S5 [file mBio.01488-20-sf005.pdf]

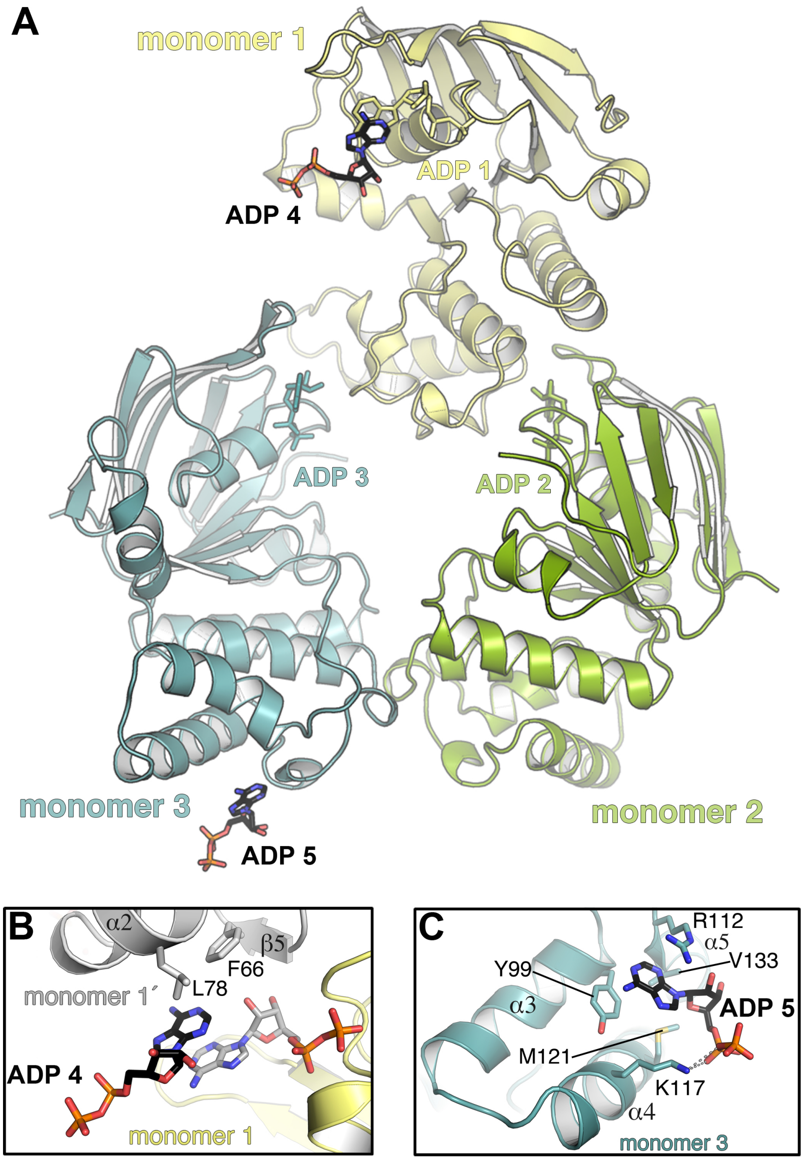

Fig. S5. (**A**) FtsE monomer arrangement in the asymmetric unit of P 2_1 crystals. The protein is depicted in ribbon, each monomer (M1-M3) colored differently. ADP is depicted in gray sticks. (**B**) and (**C**) Detailed view of stabilization of the additional ADP (ADP4 and ADP5) molecules found in the P2_1 crystal. Protein is represented in cartoon with relevant residues labeled and shown in capped sticks. ADP4 (panel B) makes a stacking interaction through their adenine purine rings with the ADP molecule bound to M1. A symmetry-related chain (monomer 1 ´, colored in gray) contributes to the stabilization of ADP4 through hydrophobic interactions mediated by L78 and F66. ADP5 (panel C) is found in a cavity formed by three monomers in the crystal and establishing main interactions with M3. The purine adenine ring is sandwiched between Y99 and R112 and the dinucleotide is further stabilized by hydrophobic interaction with M121 and V133 and polar interactions with K117.
