# Supplementary material for: Structural Characterization of the Essential Cell Division Protein FtsE and Its Interaction with FtsX in Streptococcus pneumoniae
Source: mBio. 2020 Sep 1;11(5):e01488-20. doi: 10.1128/mBio.01488-20 (PMC7468199; doi:10.1128/mBio.01488-20)
Supplement: FIG S6 [file mBio.01488-20-sf006.pdf]

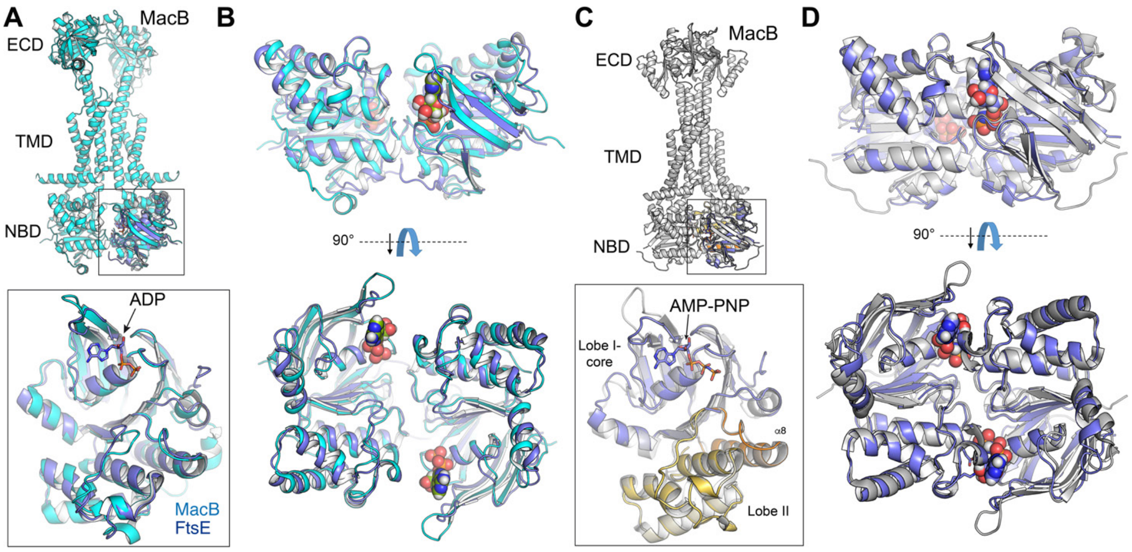

Fig. S6. (**A**, **B**) Structural model of FtsE dimer in the ADP state. (**A**) Model was created by superposition of the FtsE:ADP complex (monomer P 1, colored in dark blue) onto the nucleotide-binding domains (NBDs) of the dimer of MacB from *A. baumannii* [colored in cyan, PDB 5GKO]. Superposition presents a *rmsd* value of 1.0 Å for 174 Cα atoms. ECD, extracellular domain; TMD, transmembrane domain. Lower panel shows a detailed view of the superposition of FtsE:ADP complex onto NBD of MacB. The orientation is similar to Figure 2B (right panel). ADP is depicted in capped sticks. (**B**) FtsE:ADP dimer shown in dark blue cartoon displayed in two orientations at 90° of each other. Structural superposition with both NBDs of the dimer of MacB from *A.baumannii* (cyan cartoon) is also shown. ADP from each FtsE monomer is shown as spheres. (**C**, **D**) Structural model of FtsE dimer in the ATP-bound state. (**C**) Model was created by superposition of FtsE:AMP-PNP complex (M1 monomer colored in dark blue) onto the nucleotide-binding domains (NBDs) of the dimer of MacB:ATP from *A. actinomycetemcomitans* [colored in gray, PDB 5LJ6]. ECD, extracellular domain; TMD, transmembrane domain. Lower panel shows a detailed view of the boxed area from the upper panel comprising one single NBD. A composite model in which FtsE was fragmented in three subdomains; lobe I-core (colored in dark blue) including residues 1-86, 159-164 and 191-228; lobe II (colored in yellow) including residues 87-158 and α-helix 8 (colored in orange) and including residues 165-190. Superposition of each region onto MacB:ATP structure presents *rmsd* values of 0.8 Å for 125 Cα atoms in lobe I-core, 0.78 Å for 64 Cα atoms in lobe II and 0.5 Å for 26 Cα atoms in α-helix 8. The orientation is similar to Figure 2B (right panel). ADP is depicted in capped sticks. (**D**) FtsE dimer model in the ATP-bound state shown in dark blue cartoon displayed in two orientations at 90° of each other. Structural superposition with both NBDs of the dimer of MacB from *A.baumannii* (gray cartoon) is also shown. AMP-PNP from each FtsE monomer is shown as spheres.
