# Supplementary material for: Structural Characterization of the Essential Cell Division Protein FtsE and Its Interaction with FtsX in Streptococcus pneumoniae
Source: mBio. 2020 Sep 1;11(5):e01488-20. doi: 10.1128/mBio.01488-20 (PMC7468199; doi:10.1128/mBio.01488-20)
Supplement: FIG S7 [file mBio.01488-20-sf007.pdf]

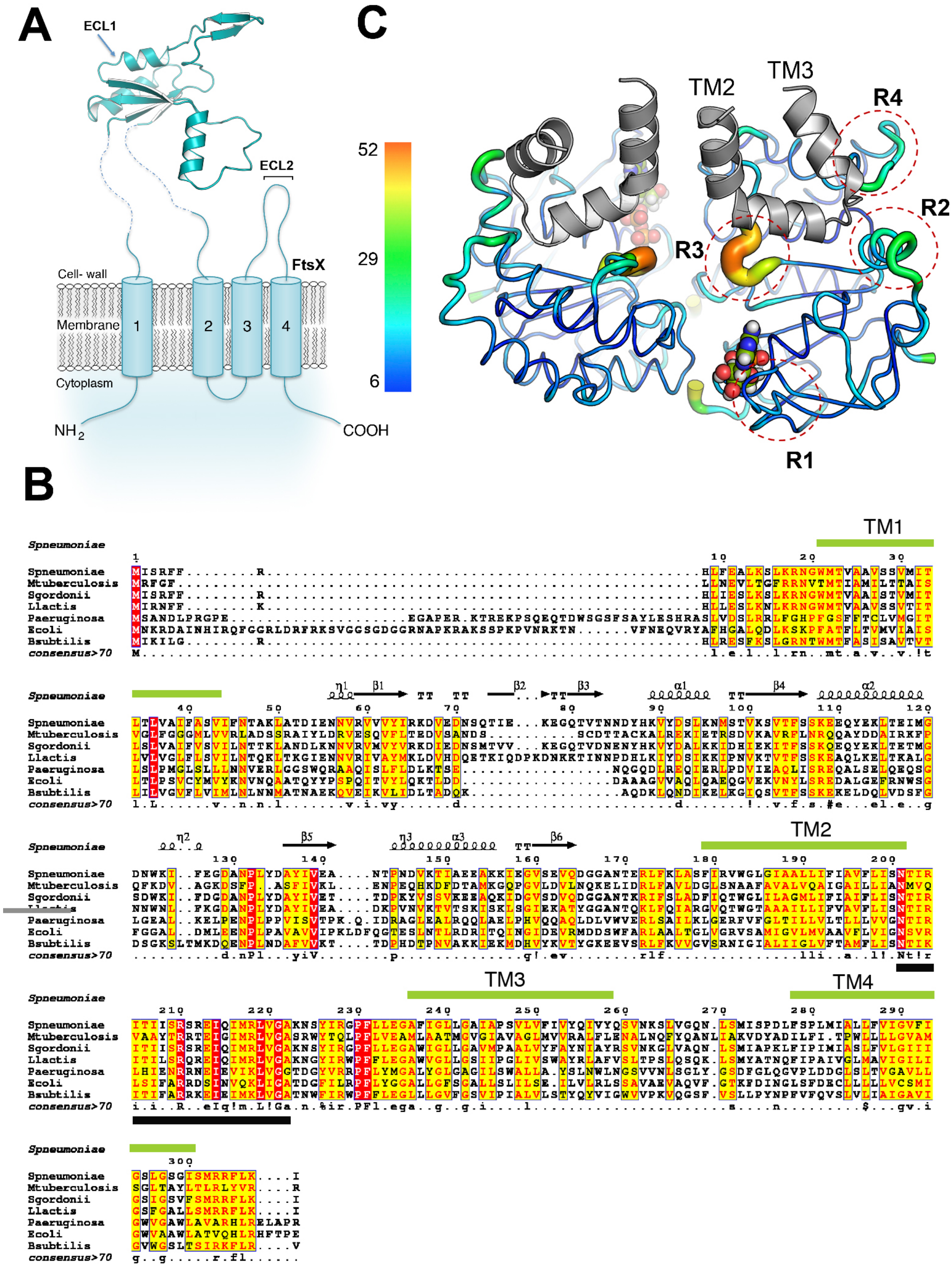

Fig. S7. (**A**) Topology of FtsX from *S. pneumoniae*. Transmembrane regions are numbered from 1 to 4. ECL, extracellular loop. ECL1 is displayed in cyan cartoon [PDB 6HFX]. (**B**) Sequence alignment among FtsX proteins belonging to different species. Alignment was carried out using T-Coffee (2) and drawn with ESPript (1). Secondary structure elements of FtsX ECL1 [PDB 6HFX] are indicated and numbered. They are displayed on the top of sequence blocks. Alpha and 3_{10} helices are represented by squiggles labelled α and η, respectively. Strands are represented by arrows. Amino acids marked with red or yellow boxes indicate sequence identity or similarity, respectively. Sequence gaps are indicated by *dashes*. FtsX sequence

corresponding to the cytoplasmic loop is indicated underlined with a thick black line. Transmembrane helices predicted with TMHMM server (http://www.cbs.dtu.dk/services/TMHMM/) are indicated with a green thick line running along the top of the sequence alignment and labeled. (**C**) Model for the FtsE dimer in the ADP-bound form including the coupling helices from FtsX (gray carton). FtsE monomers are displayed as cartoon putty showing a variable tube representation of the Cα trace in which the thickness of the tube corresponds with higher B-factor values. FtsE dimer has been colored according to the B-factor distribution. Several regions on one of the FtsE monomers are indicated (R1, R2, R3 and R4; see main text for details). TM1 and TM2: FtsX transmembrane region 1 and 2, respectively. ADP is depicted in spheres. A similar result was obtained with the FtsE model in the ATP-bound form (not shown).

References

1. Gouet P, Courcelle E, Stuart DI, Metoz F. 1999. ESPript: analysis of multiple sequence alignments in PostScript. Bioinformatics1999/05/13. 15:305–308.
2. Poirot O, Suhre K, Abergel C, O'Toole E, Notredame C. 2004. 3DCoffee@igs: a web server for combining sequences and structures into a multiple sequence alignment. Nucleic Acids Res2004/06/25. 32:W37-40.
