# Supplementary material for: Structural Characterization of the Essential Cell Division Protein FtsE and Its Interaction with FtsX in Streptococcus pneumoniae
Source: mBio. 2020 Sep 1;11(5):e01488-20. doi: 10.1128/mBio.01488-20 (PMC7468199; doi:10.1128/mBio.01488-20)
Supplement: TABLE S1 [file mBio.01488-20-st001.pdf]

Table S1

Table S1. ***S. pneumoniae*** **R6 strains used in the present study.**

Strain	Relevant genotype	Source
RH425	Δ*comA::ermAM* and streptomycin resistant; Ery^R, Sm^R	(1)
RH426	Δ*comA::ermAM* and contains Janus; Ery^R, Kan^R	(1)
SPH131	Δ*comA*, P1::P_{comR}::*comR*, P_{comX}::Janus; Ery^R, Kan^R	(2)
ds312	Δ*comA*, P1::P_{comR}::*comR*, P_{comX}::*ftsX*; Ery^R, Sm^R	This study
ds314	Δ*comA*, P1::P_{comR}::*comR*, P_{comX}::*ftsX*, Δ$ftsX_{wt}$::Janus; Ery^R, Kan^R	This study
ds751	Δ*comA*, P1::P_{comR}::*comR*, P_{comX}::*ftsX*, $ftsX^{E213K}$; Ery^R, Sm^R	This study
ds753	Δ*comA*, P1::P_{comR}::*comR*, P_{comX}::*ftsX*, $ftsX^{L219K}$; Ery^R, Sm^R	This study
ds754	Δ*comA*, P1::P_{comR}::*comR*, P_{comX}::*ftsX*, $ftsX^{V220K}$; Ery^R, Sm^R	This study
ds761	Δ*comA*, P1::P_{comR}::*comR*, P_{comX}::*ftsX*, $ftsX^{I216K}$; Ery^R, Sm^R	This study
ds768	Δ*comA*, P1::P_{comR}::*comR*, P_{comX}::*ftsX*, *ftsX-3xflag*; Ery^R, Sm^R	This study
ds769	Δ*comA*, P1::P_{comR}::*comR*, P_{comX}::*ftsX*, $ftsX^{E213K}$*-3xflag*; Ery^R, Sm^R	This study
ds770	Δ*comA*, P1::P_{comR}::*comR*, P_{comX}::*ftsX*, $ftsX^{I216K}$*-3xflag*; Ery^R, Sm^R	This study
ds771	Δ*comA*, P1::P_{comR}::*comR*, P_{comX}::*ftsX*, $ftsX^{L219K}$*-3xflag*; Ery^R, Sm^R	This study
ds772	Δ*comA*, P1::P_{comR}::*comR*, P_{comX}::*ftsX*, $ftsX^{V220K}$*-3xflag*; Ery^R, Sm^R	This study

References:

1. Johnsborg O, Håvarstein LS. 2009. Pneumococcal LytR, a protein from the LytR-CpsA-Psr family, is essential for normal septum formation in Streptococcus pneumoniae. J Bacteriol2009/07/08. 191:5859–5864.
2. Berg KH, Biørnstad TJ, Straume D, Håvarstein LS. 2011. Peptide-regulated gene depletion system developed for use in Streptococcus pneumoniae. J Bacteriol2011/08/02. 193:5207–5215.
