# Supplementary material for: Structural Characterization of the Essential Cell Division Protein FtsE and Its Interaction with FtsX in Streptococcus pneumoniae
Source: mBio. 2020 Sep 1;11(5):e01488-20. doi: 10.1128/mBio.01488-20 (PMC7468199; doi:10.1128/mBio.01488-20)
Supplement: TABLE S2 [file mBio.01488-20-st002.pdf]

Table S2

Table S2. Primers used in the present study.

Primer name	Sequence 5'→3'	Source
**Primers used to amplify 6xhis-TEV-FtsE**		
Ftse1	ACATAGAATTCGGAGAACCTGTACTTCCAAGGTTCAATTATTGAAATGAG	This study
Ftse2	ACATAAACGTTCTAATCATCGTATCCATACTCTCCTTTTGATTCGTC	This study
**Primers used to amplify Janus**		
Kan484.F	GTTTGATTTTTAATGGATAATGTG	(1)
RpsL41.R	CTTTCCTTATGCTTTTGGAC	(1)
**Primers used to construct P_{comX}-*ftsX***		
khb31	ATAACAAATCCAGTAGCTTTGG	(2)
khb33	TTTCTAATATGTAACTCTTCCCAAT	(2)
khb34	CATCGGAACCTATACTCTTTTAG	(2)
khb36	TGAACCTCCAATAATAAATATAAAT	(2)
ds313	ATTTATATTTATTATTGGAGGTTCAATGATTAGTAGATTTTTTCGCC	This study
ds314	ATTGGGAAGAGTTACATATTAGAAACTAAATCTTCAAGAATCGGCG	This study
**Primers used to construct Δ*ftsX*$_{wt}$::Janus**		
ds307	GGAGGTAGATTCTCTCAAAGG	This study
ds308	CACATTATCCATTAAAAATCAAACCTAATCATCGTATCCATACTCTC	This study
ds309	GTCCAAAAGCATAAGGAAAGGTAAAATAGCTGCTTTTATGAGG	This study
ds310	TTATTGATACCTGTTTGAACTAC	This study
**Primers used to make *ftsX*^{E213K}**		
ds608	TTTGCGACTGCGGGAAATAATGG	This study
ds609	CCATTATTTCCCGCAGTCGCAAAATTCAAATCATGCGCTTGGTC	This study
**Primers used to make *ftsX*^{I216K}**		
ds610	TTTTTGAATTTCGCGACTGCGGG	This study
ds611	CCCGCAGTCGCGAAATTCAAAAAATGCGCTTGGTCGGAGCTAA	This study
**Primers used to make *ftsX*^{L219K}**		
ds612	TTTGCGCATGATTTGAATTTCGCG	This study
ds613	CGCGAAATTCAAATCATGCGCAAAGTCGGAGCTAAAAACAGTTATATC	This study
**Primers used to make *ftsX*^{V220K}**		
ds614	TTTCAAGCGCATGATTTGAATTTCG	This study
ds615	CGAAATTCAAATCATGCGCTTGAAAGGAGCTAAAAACAGTTATATCC	This study
**Primers used to Flag-tag *ftsX***		
ds643	GATTATAAAGATCATGATGGTGATTATAAAGATCATGATATTGATTATAAAGA TGATGATGATAAATAGGTAAAATAGCTGCTTTTATGAGG	This study
ds644	TCACCATCATGATCTTTATAATCAATCTTCAAGAATCGGCGCATGG	This study
**Primers used for *ftsX* sequencing**		
ds616	AATCTCTTGGAACGGATTAACC	This study
ds617	TAGTGCGCCAAATTTCAAGTTC	This study

References:

1. Johnsborg O, Eldholm V, Bjørnstad ML, Håvarstein LS. 2008. A predatory mechanism dramatically increases the efficiency of lateral gene transfer in *Streptococcus pneumoniae* and related commensal species. Mol Microbiol2008/05/20. 69:245–253.
2. Berg KH, Biørnstad TJ, Straume D, Håvarstein LS. 2011. Peptide-regulated gene depletion system developed for use in *Streptococcus pneumoniae*. J Bacteriol2011/08/02. 193:5207–5215.
